# Metaproteomics reveals methyltransferases implicated in dichloromethane and glycine betaine fermentation by ‘*Candidatus* Formimonas warabiya’ strain DCMF

**DOI:** 10.3389/fmicb.2022.1035247

**Published:** 2022-12-07

**Authors:** Sophie I. Holland, Xabier Vázquez-Campos, Haluk Ertan, Richard J. Edwards, Michael J. Manefield, Matthew Lee

**Affiliations:** ^1^Water Research Centre, School of Civil and Environmental Engineering, University of New South Wales, Sydney, NSW, Australia; ^2^School of Biotechnology and Biomolecular Sciences, University of New South Wales, Sydney, NSW, Australia; ^3^School of Chemical Engineering, University of New South Wales, Sydney, NSW, Australia; ^4^Department of Molecular Biology and Genetics, Istanbul University, Istanbul, Turkey

**Keywords:** dichloromethane, anaerobic dechlorination, methyltransferase, glycine betaine, Wood–Ljungdahl pathway, metaproteomics, subsurface

## Abstract

Dichloromethane (DCM; CH_2_Cl_2_) is a widespread pollutant with anthropogenic and natural sources. Anaerobic DCM-dechlorinating bacteria use the Wood–Ljungdahl pathway, yet dechlorination reaction mechanisms remain unclear and the enzyme(s) responsible for carbon-chlorine bond cleavage have not been definitively identified. Of the three bacterial taxa known to carry out anaerobic dechlorination of DCM, ‘*Candidatus* Formimonas warabiya’ strain DCMF is the only organism that can also ferment non-chlorinated substrates, including quaternary amines (i.e., choline and glycine betaine) and methanol. Strain DCMF is present within enrichment culture DFE, which was derived from an organochlorine-contaminated aquifer. We utilized the metabolic versatility of strain DCMF to carry out comparative metaproteomics of cultures grown with DCM or glycine betaine. This revealed differential abundance of numerous proteins, including a methyltransferase gene cluster (the *mec* cassette) that was significantly more abundant during DCM degradation, as well as highly conserved amongst anaerobic DCM-degrading bacteria. This lends strong support to its involvement in DCM dechlorination. A putative glycine betaine methyltransferase was also discovered, adding to the limited knowledge about the fate of this widespread osmolyte in anoxic subsurface environments. Furthermore, the metagenome of enrichment culture DFE was assembled, resulting in five high quality and two low quality draft metagenome-assembled genomes. Metaproteogenomic analysis did not reveal any genes or proteins for utilization of DCM or glycine betaine in the cohabiting bacteria, supporting the previously held idea that they persist *via* necromass utilization.

## Introduction

Dichloromethane (DCM; CH_2_Cl_2_) is a widespread compound with a range of natural and anthropogenic sources. The latter accounts for 70% of the ~900 Gg DCM produced annually, while the former consists primarily of oceanic sources and biomass burning (Gribble, 2010). As a result of extensive industrial use, substantial DCM contamination in the environment continues to increase (Shestakova and Sillanpää, 2013; Hossaini et al., 2015; Leedham Elvidge et al., 2015), posing a threat to human and environmental health (Agency for Toxic Substances and Disease Registry, 2000; Hossaini et al., 2017). The compound is a priority pollutant in Europe (European Parliament Council of the European Union, 2013), the US (United States Environmental Protection Agency, 1977), and China (Ministry of Environmental Protection (MEP), 2017). Being denser than water, DCM that escapes into groundwater migrates downwards into anoxic zones. Despite this, microbial transformation of DCM in anoxic environments remains poorly understood, with only three organisms known to conserve energy *via* anaerobic DCM metabolism. *Dehalobacterium formicoaceticum* strain DMC, the only one of these in pure culture, ferments DCM to formate and acetate (Mägli et al., 1996). ‘*Candidatus* Dichloromethanomonas elyunquensis’ strain RM mineralizes DCM to H_2_ and CO_2_ (Kleindienst et al., 2017; Chen et al., 2020), while ‘*Candidatus* Formimonas warabiya’ strain DCMF ferments DCM to acetate (Holland et al., 2021). All three bacteria are members of the Peptococcaceae family and use the Wood–Ljungdahl pathway for DCM metabolism in varying ways (Mägli et al., 1998; Chen et al., 2017; Holland et al., 2019; Kleindienst et al., 2019; Holland et al., 2021).

Cell suspension assays with *D. formicoaceticum* suggested that DCM dechlorination was corrinoid-dependent (Mägli et al., 1996). Recent genomic and proteomic analysis of ‘*Ca.* Dichloromethanomonas elyunquensis’ and *D. formicoaceticum* highlighted a cluster of corrinoid-dependent methyltransferase genes thought to be involved in DCM catabolism – the *mec* cassette – and identified a homologous cassette in the strain DCMF genome (Kleindienst et al., 2019; Murdoch et al., 2022). Notably, ‘*Ca.* Dichloromethanomonas elyunquensis’ also encodes and expresses reductive dehalogenases in the presence of DCM (Kleindienst et al., 2019), which are absent from the other two organisms’ genomes (Chen et al., 2017; Holland et al., 2019). However, ‘*Ca.* Dichloromethanomonas elyunquensis’ and *D. formicoaceticum* are obligate DCM degraders, and demonstrably unable to metabolize any other chlorinated methanes (Mägli et al., 1996; Kleindienst et al., 2017). In light of this, as well as the difference in end products and previous dual carbon-chlorine isotope analyses (Chen et al., 2018), the three DCM-degrading bacteria are hypothesized to utilize differing dechlorination mechanisms, but these are not yet fully understood.

The strain DCMF genome encodes an abundance of predicted corrinoid-dependent methyltransferase genes (Holland et al., 2019), including 82 in the MttB superfamily, which contains methylamine and quaternary amine methyltransferases, as well as many of uncharacterized substrate specificity (Ferguson and Krzycki, 1997; Ticak et al., 2014). Corrinoid-dependent methyltransferase systems are typically found in methanogenic archaea and homoacetogenic bacteria and are comprised of three main components: a methyltransferase I (MTI), methyltransferase II (MTII), and cognate corrinoid protein (CoP). MTI transfers a methyl group from the substrate onto the CoP, from which MTII transfers it to the final receiving compound, typically coenzyme M in methanogens or tetrahydrofolate (THF) in acetogenic bacteria (reviewed in Ragsdale, 2008). A reductive activator of corrinoid-dependent enzymes (RACE) protein may also be required to reactivate the corrinoid cofactor (Ragsdale, 2008), although this is unlikely to be required every reaction cycle (Drummond et al., 1993; Menon and Ragsdale, 1999). In bacteria, these methyltransferases can demethylate chloromethane (Vannelli et al., 1999), methanol (van der Meijden et al., 1984), quaternary amines (Ticak et al., 2014; Picking et al., 2019; Kountz et al., 2020), and methoxylated aromatic compounds such as vanillate (Kaufmann et al., 1997).

Strain DCMF is a methylotrophic acetogen that ferments a number of these compounds – methanol, choline (*N*,*N*,*N*-trimethylethanolamine), and glycine betaine (*N*,*N*,*N*-trimethylglycine) – as well as DCM (Holland et al., 2021). Glycine betaine is a quaternary amine with substantial environmental roles, including widespread use as an osmoprotectant across all domains of life (Beers, 1967; Larher et al., 1982; Csonka, 1989). Strain DCMF is the dominant lineage in enrichment culture DFE, although its relative abundance varies from ~7% to 75% throughout a single substrate pulse with DCM or glycine betaine (Holland et al., 2021). Despite this, previous investigation of the strain DCMF genome, growth curve mass balances, and strain DCMF-free enrichment cultures have all suggested that it is highly unlikely the cohabitant bacteria are involved in primary substrate metabolism (Holland et al., 2021). However, it remains unclear how they persist in culture DFE. Based on physiological and genomic information, strain DCMF demethylates glycine betaine in a stepwise manner to *N*,*N*-dimethylglycine and then sarcosine (*N*-methylglycine), before being reductively cleaving sarcosine to monomethylamine and acetate. Methyltransferases likely catalyze methyl group transfer from glycine betaine and dimethylglycine onto THF and the resulting methyl-THF enters the Wood–Ljungdahl pathway resulting in acetate production (Holland et al., 2019, 2021). While glycine betaine methyltransferases have previously been characterized in *Desulfitobacterium hafniense* (Ticak et al., 2014) and *Acetobacterium woodii* (Lechtenfeld et al., 2018), there is only a single proteomic study (in *Sporomusa ovata* strain An4) investigating this enzyme (Visser et al., 2016).

Here, we aimed to determine the enzymes involved in DCM and glycine betaine metabolism in strain DCMF *via* comparative metaproteomics of enrichment culture DFE. This addressed knowledge gaps surrounding the proteins involved in DCM dechlorination and glycine betaine fermentation in this organism, and more broadly in anoxic subsurface environments. Methyltransferase systems were implicated in both metabolisms, providing gene targets for further study.

## Materials and methods

### Metagenomic analysis

To assemble the culture DFE metagenome, a previously described set of PacBio-sequenced, non-redundant (NR) contigs that did not map to the strain DCMF genome (“NR contaminants”; Holland et al., 2019) were assembled using Metaflye v2.7.1 (Kolmogorov et al., 2020) with parameters --pacbio-raw --genome-size 20 m --meta --plasmids. The assembled contigs were frameshift-corrected using DIAMOND v0.9.31 (Buchfink et al., 2014) and MEGAN Community Edition v6.19.9 (Huson et al., 2016), as previously described (Arumugam et al., 2019).

“NR contaminants” reads, as well as those from previous Illumina sequencing efforts of culture DFE (SRA accession number SRR5179547; Holland et al., 2019), were mapped to the frameshift-corrected contigs using BBMap v38.51 and Bowtie2 v2.3.5.1, respectively. Taxonomy was assigned to contigs using Kaiju v1.7.2. This information was parsed into anvi’o (installed from the master Github repository on 1 October 2020; Eren et al., 2021) for manual binning. CheckM v1.1.3 (Parks et al., 2015) was used to assess completeness and contamination of each bin and taxonomy was assigned with GTDB-tk v2.0.0 (Chaumeil et al., 2019) against release R07-RS207. Gene calling was performed with Prokka v1.14.5 (Seemann, 2014). Predicted proteins were annotated with orthologous groups and KEGG database information using EggNOG-mapper v2.0 (database v5.0; Huerta-Cepas et al., 2019) and with subcellular localization using PSORTb v3.0.2 (Yu et al., 2010).

Metabolic pathways in the MAGs were determined *via* BlastKOALA (Kanehisa et al., 2016), dbCAN2 (Zhang et al., 2018) predicted Carbohydrate Active enZymes (CAZymes) and hydrogenase catalytic subunits were classified using HydDB (Søndergaard et al., 2016).

### Cultures for metaproteomics

Culture DFE was grown in anaerobic, defined minimal mineral salts medium as previously described (Holland et al., 2019). The medium was buffered to pH 6.8–7.0 *via* addition of NaHCO_3_ (2.5 g L^-1^) and sparging with N_2_:CO_2_ (4,1). For metaproteomic analysis, 200 ml cultures were amended with either DCM (2 mM) or glycine betaine (5 mM; *n* = 6 for each). Cultures were incubated statically at 30°C in the dark. DCM was quantified by GC-FID and glycine betaine by LC–MS/MS (Holland et al., 2021).

Cells were harvested from cultures when ~80% of the substrate was depleted (Supplementary Figure S1). Biomass from two culture flasks (i.e., 400 ml total) were combined to produce triplicate samples for metaproteomic analysis of each substrate condition. Strain DCMF cells were enumerated in cultures *via* quantitative real-time PCR (qPCR) of the strain DCMF 16S rRNA gene with primers Dcm775F/Dcm930R, as previously described (Holland et al., 2019). Total bacterial 16S rRNA genes were quantified similarly, with primers Eub1048/Eub1194 (Holland et al., 2021). Strain DCMF 16S rRNA gene copy numbers were converted to cell numbers by dividing by four (the number of 16S rRNA gene copies in the strain DMCF genome).

Cultures were centrifuged at 8,000 × *g* at 4°C for 30 min and then resuspended in 120 μl protein extraction buffer (50 mM 3-(N-morpholino)propanesulfonic acid [pH 7], 4% sodium dodecylsulfate, 50 mM NaCl, 100 μM EDTA, 100 μM MgCl_2_). Mixtures were transferred to 2 ml tubes containing 0.06 g glass beads (150–212 μm, Sigma, North Ryde, Australia) and a ¼” ceramic sphere (MP Bio, Seven Hills, Australia) and bead-beat at 1,800 rpm for 5 min (PowerLyzer 24 Homogenizer, Qiagen, Chandstone Centre, Australia). Tubes were centrifuged at 16,000 × *g* for 10 min and the supernatants (i.e., crude protein extracts) transferred to fresh, 1.5 ml tubes to repeat the centrifugation.

Protein yield was quantified using the Micro BCA Protein Assay Kit (Thermo Fisher Scientific, Scoresby, Australia) with crude protein extracts diluted 1:250 in MilliQ water. Bovine serum albumin was used to create a seven-point standard curve (0.5–40 μg ml^-1^).

### Filter-aided sample preparation

Filter-aided sample preparation (FASP) was used to prepare the crude protein extracts for peptide identification (Wiśniewski, 2017). Samples were diluted to a concentration of ~1 μg μl^-1^ in 50 mM NH_4_HCO_3_. A total of 15.8 μg protein from each sample was transferred to a 1.5 ml microfuge tube with 5 mM dithiothreitol and incubated at 37°C for 30 min. Samples were loaded onto Amicon Ultra-0.5 30 kDa centrifugal filter units (Merck, Bayswater, Australia) with 200 μl UA solution (8 M urea in 100 mM Tris–HCl, pH 8.5). Filters were centrifuged at 14,000 × *g* for 15 min before another 200 μl UA was added to each and the centrifugation repeated. Proteins were alkylated by addition of 100 μl iodoacetamide solution (50 mM iodoacetamide in UA) and mixing at 600 rpm for 1 min prior to incubating statically in the dark for 20 min. Filters were centrifuged at 14,000 × *g* for 10 min. UA (100 μl) was added to each filter before centrifuging at 14,000 × *g* for 15 min, twice. Then, 50 mM NH_4_HCO_3_ (100 μl) was added to each filter before centrifuging at 14,000 × *g* for 10 min; repeated twice more. Proteolytic cleavage into peptides was performed by addition of trypsin (1:100 enzyme:protein ratio) in 40 μl NH_4_HCO_3_ and mixing at 600 rpm for 1 min. Filters were incubated in a 37°C wet chamber overnight, then transferred to fresh collection tubes and centrifuged at 14,000 × *g* for 10 min. A final 20 μl NH_4_HCO_3_ was added to each filter before centrifuging at 14,000 × *g* for 10 min; this was repeated once more. Eluent was stored at -80°C.

### Peptide lysate analysis *via* LC–MS/MS

Peptide lysates were separated by nanoLC on an UltiMate™ 3,000 RSLCnano ultra performance liquid chromatograph and autosampler system (Dionex, Scoresby, Australia). Samples (2.5 μl) were concentrated and desalted onto a micro C18 precolumn (300 μm × 5 mm, Dionex) with water:acetonitrile (98:2, 0.2% TFA) at 15 μl min^-1^. After a 4 min wash the pre-column was switched (10 port UPLC valve, Valco, Houston, TX) into line with a fritless nano column (75 μm × 15 cm) containing C18AQ media (1.9 μ, 120 Å, Dr. Maisch). Peptide lysates were eluted using a linear gradient of water:acetonitrile (98:2, 0.1% formic acid) to water:acetonitrile (64:36, 0.1% formic acid) at 200 nl min^-1^ over 30 min. High voltage 2000 V was applied to low volume Titanium union (Valco) and the tip positioned ~0.5 cm from the heated capillary (*T* = 275°C) of an Orbitrap Fusion Lumos (Thermo Electron, Scoresby, Australia) mass spectrometer. Positive ions were generated by electrospray and the Fusion Lumos operated in data dependent acquisition mode. A survey scan *m/z* 350 – 1,750 was acquired (resolution = 120,000 at *m/z* 200, with an accumulation target value of 400,000 ions) and lockmass enabled (*m/z* 445.12003). Data-dependent tandem MS analysis was performed using a top-speed approach (cycle time of 2 s). MS/MS spectra were acquired by HCD (normalized collision energy = 30) fragmentation and the ion-trap was selected as the mass analyser. The intensity threshold for fragmentation was set to 25,000. A dynamic exclusion of 20 s was applied with a mass tolerance of 10 ppm.

### Metaproteomic data analysis

Mass spectra files were searched against a custom database of all predicted proteins in the DFE metagenome using MaxQuant v1.6.17.0 (Cox et al., 2014). Enzyme specificity was trypsin/P with a maximum of two missed cleavages. Fixed (carbamidomethylation of cysteine) and variable (oxidation of methionine and N terminal acetylation) modifications were selected. Minimum peptide length was seven amino acids and maximum peptide mass 4,600 Da. The mass tolerance was set to 4.5 ppm for the MS and 0.5 Da for the MS/MS. ‘LFQ’ and ‘Match between runs’ were selected. The PSM and protein False Discovery Rate (FDR) were both 0.01. The mass spectrometry and proteomics data have been deposited to the ProteomeXchange Consortium *via* the PRIDE partner repository (Perez-Riverol et al., 2019) with the dataset identifier PXD037334.

Statistical analysis of the MaxQuant output was performed in Perseus v1.6.13.0 (Tyanova et al., 2016). Proteins identified by site, reverse sequences, only one unique peptide, and potential contaminants were removed. Proteins were filtered to retain only those present in all three replicates of at least one substrate condition. Label free quantitative (LFQ) intensities were log_2_ transformed and missing values were imputed from a Gaussian distribution (down shift 1.8, width 0.3, relative to the standard deviation of each column). Triplicate-averaged values were Z-score transformed within each column to determine protein abundance relative to overall expression with each substrate. Only proteins in the ‘Majority Protein ID’ column were considered present, i.e., those where all proteins listed in a group had at least half the peptides that the leading protein had. Where >1 proteins were included in the ‘Majority Protein ID’ group, they were included for analysis and are listed separately, but marked as ‘Ambiguous’ and treated with appropriate caution in interpreting any results. Triplicate LFQ values were directly compared *via* multiple t-tests to create a volcano plot (S_0_ = 0.1, 250 randomizations, substrate grouping not preserved in randomizations). Proteins were considered differentially abundant if they had a FDR < 0.01.

## Results and discussion

### Genome-based metagenomics of culture DFE

To investigate the persistence of cohabiting bacteria in culture DFE and provide a taxa-specific platform for metaproteomic analysis, metagenomic assembly of a set of previously described (Holland et al., 2019) non-redundant, non-strain DCMF PacBio sequencing reads (“NR Contaminants”) was carried out. Sequencing details were previously reported (Holland et al., 2019) but assembly of this subset of reads was not previously attempted. A total 195,364 long reads (732,500,489 bp) assembled into 330 contigs (total size 13,752,672 bp; Supplementary Table S1). Reads were deposited in the NCBI SRA (SRX9412577).

Manual binning with anvi’o resulted in 10 bins (Supplementary Figure S2). Based on their completeness, contamination, and the presence of 16S rRNA genes, these comprise five high quality and two low quality draft metagenome-assembled genomes (MAGs; Supplementary Table S2), and three undetermined bins (“UNK-1/2/3”). Taxonomic assignment of the MAGs revealed unclassified lineages of *Bacteroidales* (henceforth referred to as “DFE-LEN”), *Ignavibacteria* (“DFE-IGN”), *Cupidesulfovibrio* (“DFE-NIT”; a recently proposed novel genus based on *Desulfovibrio oxamicus* and *Desulfovibrio termitidis* (Wan et al., 2021)), *Synergistales* (“DFE-SYN”), and *Rectinema* (“DFE-TRE1”; Table 1). Taxonomic assignment was not performed for bins below 50% completeness (“DFE-BAC” and “DFE-TRE2”). High-quality MAGs were deposited in NCBI (GenBank accessions in Supplementary Table S2).

**Table 1:** Overview of the MAGs assembled from the DFE culture metagenome.

Bin	Taxonomy^a^	Size (bp)	GC Content (%)	CDS	Coverage depth^b^	Completeness (%)^c^	Contamination (%)^c^
DFE-BAC	Not included as genome completeness is <50%.	1,611,616	49.93	1,590	9	29.19	0.25
DFE-IGN	d__Bacteria;p__Bacteroidota;c__Ignavibacteria; o__SJA-28;f__B-AR;g__CAIKZJ01; s__CAIKZJ01 sp015657505	3,237,034	43.07	2,738	40	94.81	1.93
DFE-LEN	d__Bacteria;p__Bacteroidota;c__Bacteroidia; o__Bacteroidales;f__UBA4417;g__UBA4417; s__UBA4417 sp015657475	4,408,406	44.01	3,545	23	95.43	2.69
DFE-NIT	d__Bacteria;p__Desulfobacterota_I; c__Desulfovibrionia;o__Desulfovibrionales; f__Desulfovibrionaceae;g__Cupidesulfovibrio; s__Cupidesulfovibrio sp000226255	3,820,024	67.18	3,142	16	86.41	3.13
DFE-SYN	d__Bacteria;p__Synergistota;c__Synergistia; o__Synergistales;f__79-D21;g__79-D21; s__79-D21 sp015657435	2,678,986	59.45	2,510	34	94.58	0.15
DFE-TRE1	d__Bacteria;p__Spirochaetota;c__Spirochaetia; o__Treponematales;f__UBA8932;g__Rectinema; s__Rectinema sp015657395	3,034,080	55.30	2,802	10	91.95	0
DFE-TRE2	Not included as genome completeness is <50%.	1,908,291	55.91	1,786	21	43	3.45

a Determined by GTDB-Tk v2.0.0 release R07-RS207.

b Based on the PacBio reads only.

c Determined by CheckM.

Annotation of the bins resulted in 1,590 coding sequences (CDS) in the smallest and least complete MAG (DFE-BAC) and 3,545 CDS in the largest (DFE-LEN; Table 1, full annotation available in Supplementary Table S3). DFE-IGN was omitted from further analysis as it was lost from the culture through subcultivation in the 3 years (~10 transfers) between PacBio sequencing and metaproteomic analysis.

### The metaproteome of culture DFE

**Figure 1:**
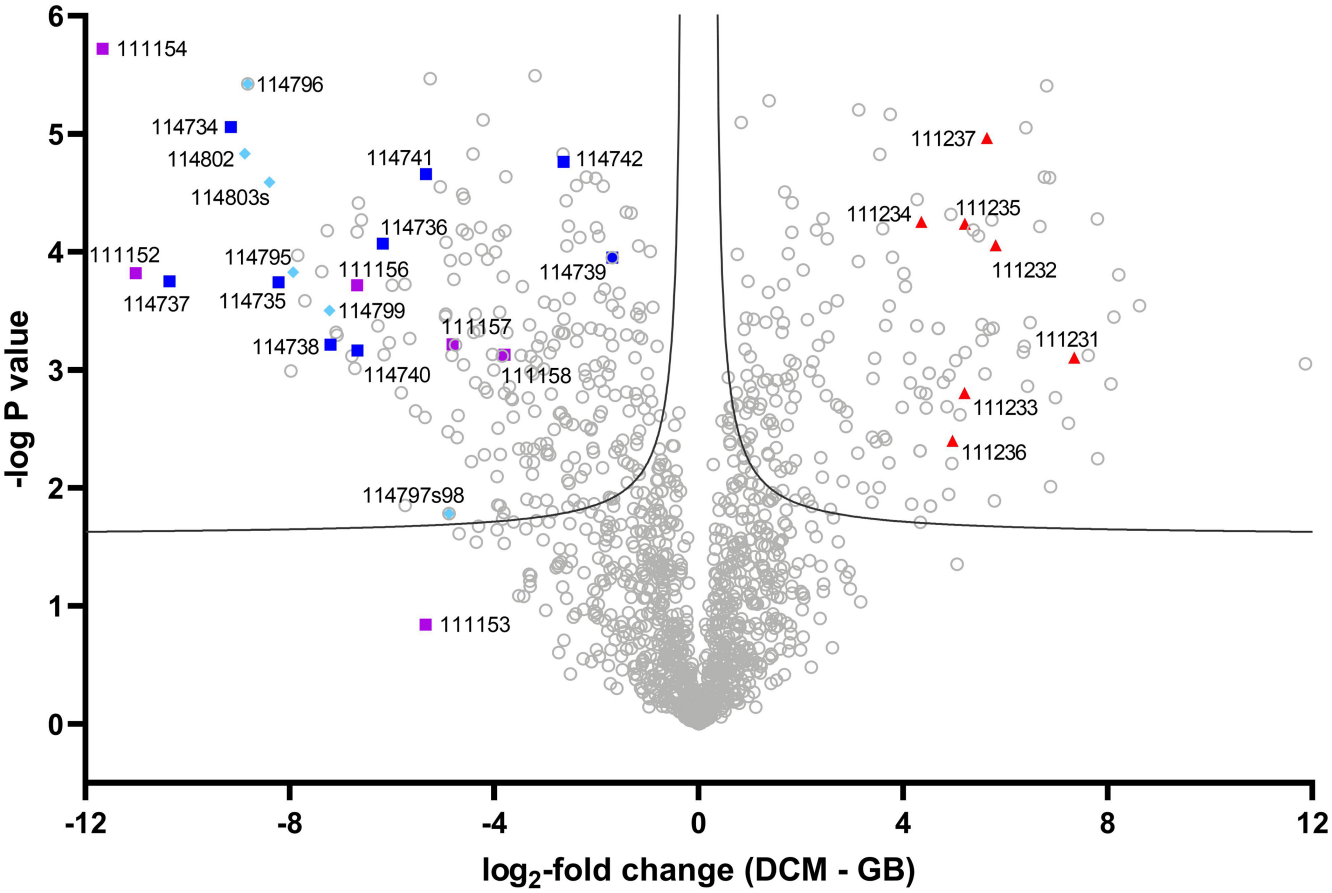
Volcano plot of strain DCMF protein expression with DCM and glycine betaine. Log_2_-fold change (LFC) in abundance is shown as the difference between DCM and glycine betaine label-free quantitative intensity. A false discovery rate of 0.01 was the significance boundary, indicated by two lines on the graph. Proteins in the DCM-associated *mec* cassette are labeled as red triangles, those from the complete glycine betaine methyltransferase gene cluster by dark blue squares, those in the incomplete glycine betaine and/or dimethylglycine methyltransferase gene cluster by purple squares, and those from the sarcosine reductase gene cluster by light blue diamonds. IMG gene loci (prefaced by ‘Ga018035_’) are indicated for these proteins of interest. A full list of all significantly differentially abundant proteins is included in Supplementary Table S5.

Label-free quantitative metaproteomic analysis of DFE cultures grown with DCM or glycine betaine identified 1,713 proteins across the two substrate conditions (Supplementary Table S4). The IMG annotation of the strain DCMF genome was used throughout (JGI genome ID 2718217647). While only those proteins present in all three replicates of at least one substrate conditions (i.e., DCM or glycine betaine) were included, missing values were imputed for all reported analyses (see Methods). Most proteins (81%) were from strain DCMF (1,384), followed by DFE-SYN (134), DFE-LEN (60), DFE-NIT (43), DFE-TRE2 (42), DFE-TRE1 (41), DFE-BAC (3), and UNK-1/2/3 (5). There were 409 significantly differentially abundant strain DCMF proteins (FDR 0.01; Figure 1; Supplementary Table S5). While the relative abundance of bacterial community members may have some bearing on the direct comparison of protein abundance between DCM- and glycine betaine-amended cultures, it is unlikely to play a large role in the data presented here for two reasons. Firstly, there was no significant difference in strain DCMF abundance between DCM vs. glycine betaine-amended cultures (either before or after duplicate culture samples were combined), as quantified by qPCR of the DCMF 16S rRNA gene (*p* > 0.05, two-tailed unpaired homoscedastic *t*-test; Supplementary Table S6). Secondly, we previously found no significant difference in the Shannon Diversity Index between culture DFE microcosms amended with different substrates (Kruskal–Wallis value of p 0.0976; Holland et al., 2021). Strain DCMF represented at least 73% relative abundance in the community when the metaproteomic samples were harvested (Supplementary Table S6).

The unique ability of strain DCMF to grow on substrates other than DCM allowed, for the first time, a statistical comparison of protein abundance in cells fermenting DCM vs. cells fermenting an alternative substrate (glycine betaine), where previous studies only examined fed vs. starvation conditions (Kleindienst et al., 2019; Murdoch et al., 2022). DCM- and glycine betaine-amended cultures were compared to determine the log_2_-fold change (LFC) of each protein. Proteins of interest were identified as those with particularly high/low LFC, as well as searches for homologs to proteins previously reported to be involved in DCM and glycine betaine metabolism in the literature (i.e., the *mec* cassette, glycine betaine methyltransferases, and glycine/betaine/sarcosine reductases).

**Figure 2:**
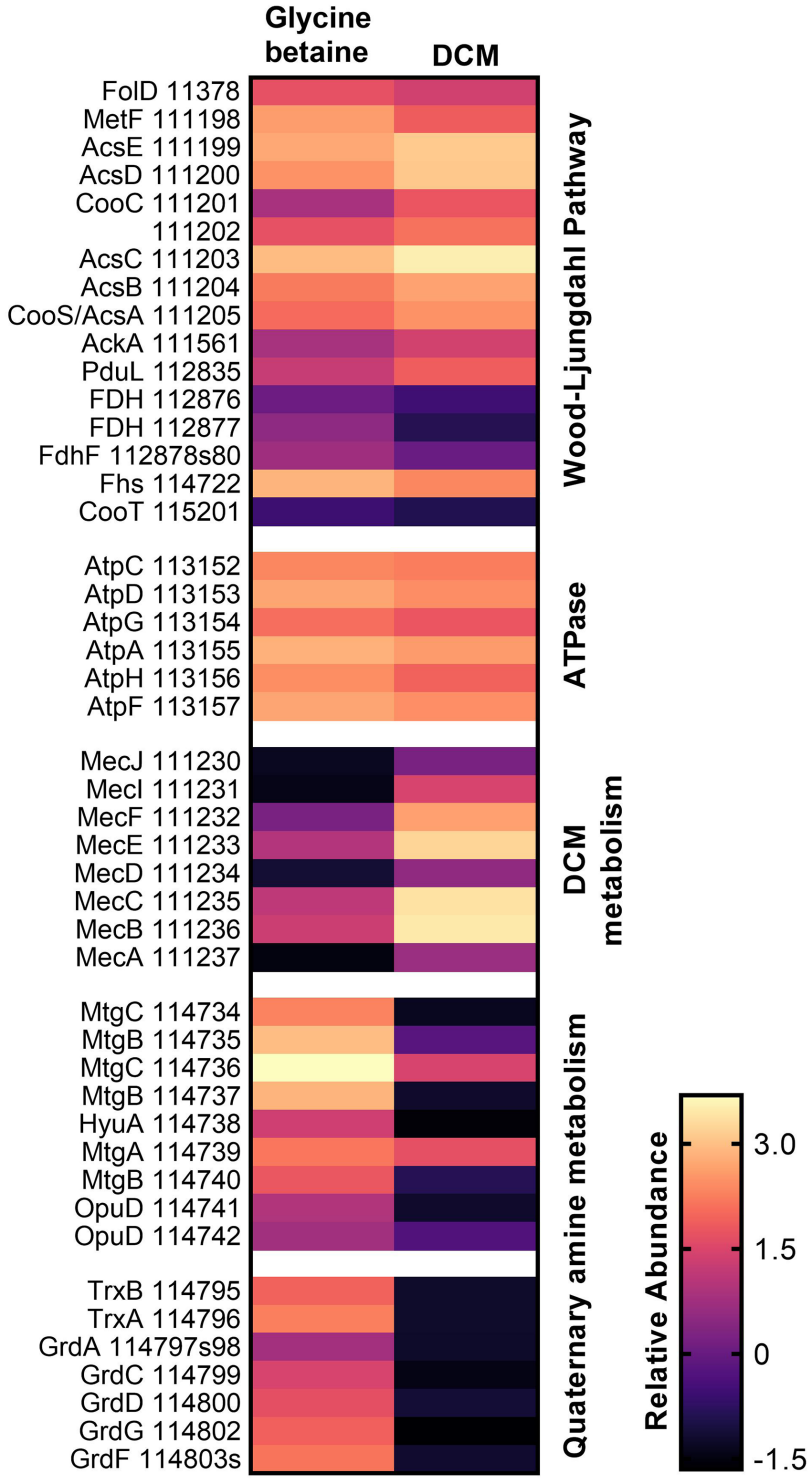
Heatmap of relative protein abundance for genes/pathways of interest in DCM and glycine betaine-amended strain DCMF cells. Proteins for the Wood–Ljungdahl pathway and a F_O_F_1_-type ATPase were highly abundant under both growth conditions, while proteins in the DCM-associated *mec* cassette were significantly more abundant in DCM-grown cells, and proteins in a putative glycine betaine methyltransferase gene cluster and a sarcosine reductase gene cluster were significantly more abundant in glycine betaine-grown cells. Rows are labeled with gene symbols and the strain DCMF IMG gene loci (each prefaced with “Ga0180325_”).

Proteins that were highly abundant under both substrate conditions were also analyzed. Within strain DCMF, this included proteins for the Wood–Ljungdahl pathway, conversion of acetyl-CoA to acetate, and six of eight subunits for an F_O_F_1_-type ATP synthase (Figure 2; Supplementary Table S4, labeled in the Pathway/Function column as “Wood–Ljungdahl Pathway” and “Energy conservation,” respectively). From the complete Rnf complex and NADH:ubiquinone reductase (complex I) encoded in the genome (Holland et al., 2019), three subunits from the former (RnfBCG, Ga0180325_113065, Ga0180325_113068, Ga0180325_113070) and 10 subunits from the latter (NuoBCDEFGHIJM, Ga0180325_115678-80, Ga0180325_11791-92, Ga0180325_11330, Ga0180325_115681-83, Ga0180325_115686) were found in the proteome (Supplementary Table S4, “Energy conservation”). Two proteins for a putative K^+^ or Na^+^-stimulated pyrophosphate-energized sodium pump (Ga0180325_113285, Ga0180325_113311) were also highly abundant with all substrates (Supplementary Table S4, “Energy conservation”). Cumulatively, this suggests that strain DCMF generates energy through a chemiosmotic mechanism as well as substrate level phosphorylation (i.e., fermentation). Furthermore, proteins for a flagellum and chemotaxis indicated that strain DCMF is motile and responds to environmental cues (Supplementary Table S4, “Flagellum” and “Chemotaxis”).

### Putative methyltransferases involved in DCM metabolism

The strain DCMF genome encodes an abundance of corrinoid-dependent methyltransferase gene components, i.e., MTI, MTII and CoPs (Holland et al., 2019). Many of these were identified in the proteome and had significantly differential abundance between DCM- and glycine betaine-amended cultures. For example, in DCM-grown cells, a protein in the monomethylamine methyltransferase mtmB superfamily (i.e., a likely MTII) had the highest LFC (Ga0180325_111810, +11.87; Figure 1; Supplementary Table S5). The *mec* cassette, which was recently suggested to be involved in anaerobic DCM dechlorination (Murdoch et al., 2022), also stood out as significantly more abundant, with an average LFC of 5.34 (Figure 2; Table 2). This eight-gene cassette includes several corrinoid-dependent methyltransferase components, a two-component regulatory system and a cation exchange protein (Table 2). Importantly for the function of these methyltransferases (and despite the presence of 50 μg L^-1^ cyanocobalamin in the culture medium) strain DCMF encodes a complete corrinoid biosynthesis pathway (Holland et al., 2019) and 14 of these 25 proteins were detected in the proteome (Supplementary Table S4, “Corrinoid biosynthesis”).

**Table 2:** List of proteins putatively involved in DCM and glycine betaine metabolism in strain DCMF.

	Protein	IMG Locus Tag	Putative function	Length (AA)	LFC^a^	-log(*p*-value)^b^
DCM metabolism	MecA	Ga0180325_111237	Two-component transcriptional regulator, histidine kinase	430	5.64	4.96
	MecB	Ga0180325_111236	Corrinoid protein	201	4.97	2.40
	MecC	Ga0180325_111235	MTI	343	5.21	4.24
	MedD	Ga0180325_111234	Two-component transcriptional regulator, receiver	272	4.36	4.25
	MecE	Ga0180325_111233	MTI, CmuA/MtaA family	337	5.2	2.81
	MecF	Ga0180325_111232	MTII	299	5.81	4.06
	MecI	Ga0180325_111231	MTI	288	7.35	3.10
	MecJ	Ga0180325_111230	Cation transporter	398	4.14	2.89
Glycine betaine demethylation	MtgC	Ga0180325_114734	Corrinoid protein	210	−9.15	5.06
	MtgB	Ga0180325_114735	Glycine betaine:corrinoid methyltransferase (MTI)	485	−8.22	3.74
	MtgC	Ga0180325_114736	Corrinoid protein	210	−6.18	4.07
	MtgB	Ga0180325_114737	Glycine betaine: corrinoid methyltransferase (MTI)	488	−10.35	3.75
	?	Ga0180325_114738	Unknown	668	−7.20	3.21
	MtgA	Ga0180325_114739	Corrinoid:tetrahydrofolate methyltransferase (MTII)	265	−1.69	3.95
	MtgB	Ga0180325_114740	Glycine betaine:corrinoid methyltransferase (MTI)	471	−6.68	3.17
	OpuD	Ga0180325_114741	Betaine/choline/carnitine family transporter	540	−5.33	4.66
	OpuD	Ga0180325_114742	Betaine/choline/carnitine family transporter	538	−2.64	4.76
Sarcosine reduction	TrxB	Ga0180325_114795	Thioredoxin reductase	407	−7.93	3.83
	TrxA	Ga0180325_114796	Thioredoxin	104	−8.82	5.42
	GrdA	Ga0180325_114797s98	Glycine/betaine/sarcosine reductase complex protein A	147	−4.89	1.78
	GrdC	Ga0180325_114799	Glycine/betaine/sarcosine reductase complex protein C	513	−7.22	3.50
	GrdD	Ga0180325_114800	Glycine/betaine/sarcosine reductase complex protein C	388	−6.91	6.34
	GrdG	Ga0180325_114802	Sarcosine reductase complex protein B	428	−8.88	4.83
	GrdF	Ga0180325_114803s	Sarcosine reductase complex protein B	436	−8.39	4.59

a log_2_-fold change in protein abundance in DCM-grown cells compared to glycine betaine-grown cells.

b All proteins listed are significantly differentially abundant.

The significant abundance of proteins in the *mec* cassette in DCM-amended strain DCMF cells provides experimental evidence supporting the hypothesis that these proteins are responsible for anaerobic dechlorination of DCM (Murdoch et al., 2022). Functional annotation of the proteins also supported previously suggested roles, that were based on expression of the *mec* cassette in *D. formicoaceticum* and ‘*Ca.* Dichloromethanomonas elyunquensis’ (Murdoch et al., 2022). Transcription of the cluster may be regulated directly in response to DCM, as the sensor histidine kinase (Ga0180325_111237, MecA) in the two-component transcriptional regulatory system harbors a PocR domain (Supplementary Table S7), which can bind DCM or other small hydrocarbons (Anantharaman and Aravind, 2005). Protein Ga0180325_111233 (MecE), classed as an MtaA/CmuA family methyltransferase (Supplementary Table S7), is the most likely candidate for the initial dechlorination of DCM. The MtaA/CmuA family contains methanol and chloromethane MTI proteins (van der Meijden et al., 1984; Vannelli et al., 1999). Chloromethane dechlorination is a methyltransfer reaction catalyzed by two subunits in aerobic methylotrophs: a fused MTI-CoP (CmuA) and an MTII (CmuB; Vannelli et al., 1998, 1999; Studer et al., 1999). A similar system is thought to operate in the anaerobe *Acetobacterium dehalogenans* (Meßmer et al., 1996; Wohlfarth and Diekert, 1997) and it is therefore feasible that MecE could act on DCM under anoxic conditions. The MtaA/CmuA protein family sits within the uroporphyrinogen decarboxylase (URO-D) superfamily, and two other proteins in the same gene cluster were also classified within this superfamily (Ga0180325_111231, MecI, and Ga0180325_111235, MecC; Supplementary Table S7). However, their role here remains unclear and as such, participation in DCM dechlorination cannot be excluded. We further hypothesize that protein Ga0180325_111232 (MecF) acts as an MTII, given that it can likely bind THF *via* a pterin-binding site (Supplementary Table S7) and may therefore catalyze the formation of 5,10-methylene-THF from DCM or a dechlorinated intermediate. Finally, protein Ga0180325_111236 (MecB) encodes a B_12_-binding site (Supplementary Table S7) and could thus act as the CoP for the proposed methyltransferase.

**Figure 3:**
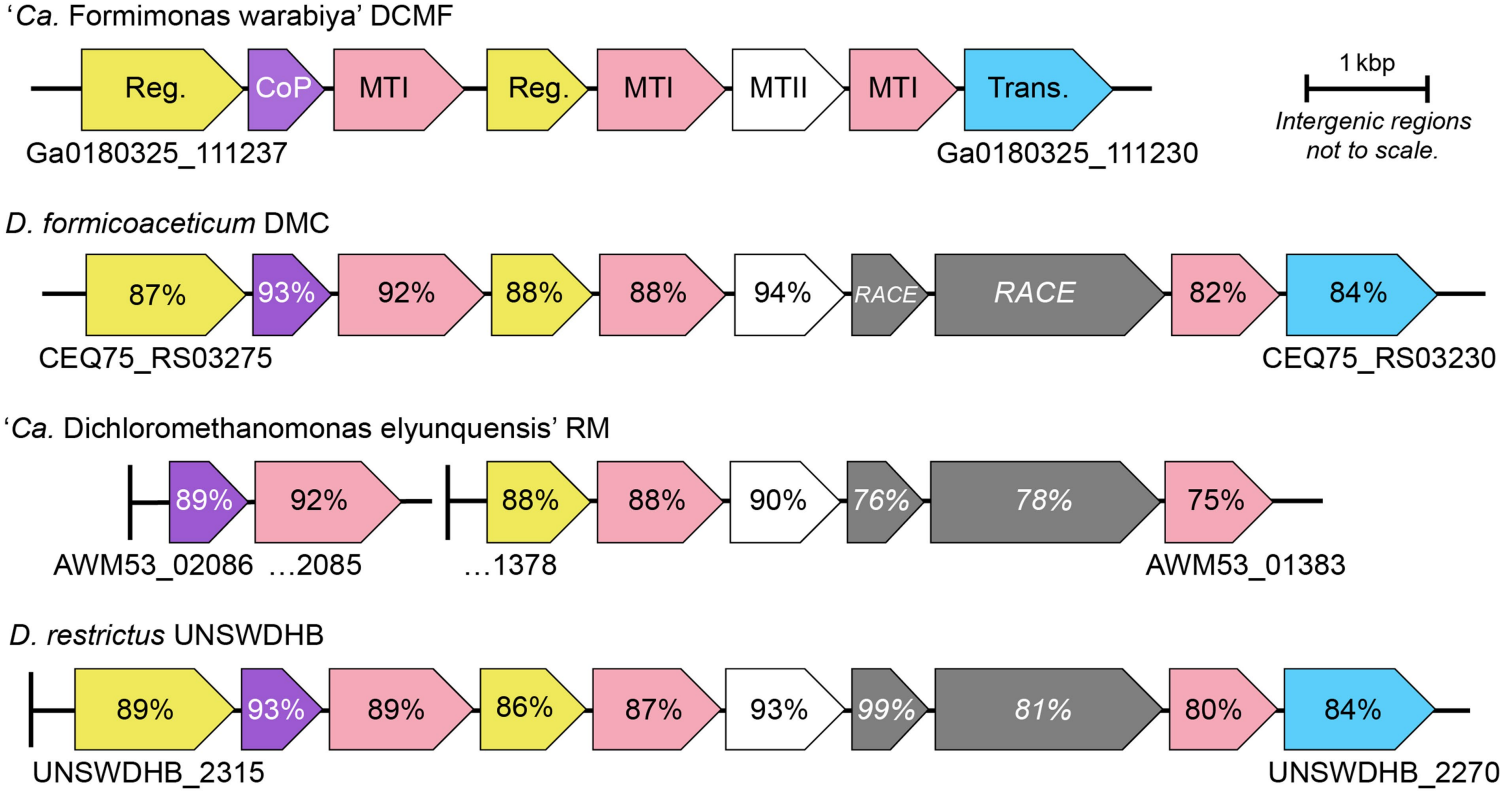
Genetic organization of the *mec* cassette in strain DCMF and other anaerobic chlorinated methane-degrading bacteria. Loci are shown below the first and last gene in each cluster. Percentage sequence identity to strain DCMF is shown for each homologous protein; percentage identity for the DUF proteins (italicized) is in relation to *D. formicoaceticum*. Vertical lines represent contig boundaries. Reg, regulator; CoP, corrinoid protein; MTI, methyltransferase I; MTII, methyltransferase II; Trans, transporter; RACE, reductive activator of corrinoid-dependent enzymes.

Full *mec* cassette homologs are encoded in DCM-fermenting bacteria *D. formicoaceticum* (CEQ75_RS03275–30) and ‘*Ca.* Dichloromethanomonas elyunquensis’ (AWM53_02086–85 and AWM53_01378-83), as well as the non-DCM-fermenting bacterium *Dehalobacter restrictus* strain UNSWDHB, which respires trichloromethane. Partial cassettes are also found in *D. restrictus* strains *CF* (which also respires trichloromethane) and DCA (which respires 1,2-dichloroethane; Murdoch et al., 2022). Within DCM-fermenting bacteria and *D. restrictus* UNSWDHB, the gene cassette is highly conserved in terms of genetic synteny and protein sequence (75–94% amino acid identity; Figure 3). Outside of the *mec* cassette homologs, the methyltransferase components share <45% amino acid identity to their closest characterized homologs (Supplementary Table S8).

Strain DCMF lacks two homologous proteins that are present in all other *mec* cassettes - MecGH (Murdoch et al., 2022). These two proteins are likely involved in reactivation of the methyltransferase corrinoid cofactor (Ferguson et al., 2009; Schilhabel et al., 2009; Price et al., 2018; Murdoch et al., 2022). It is unusual that these genes are absent from the *mec* cassette in strain DCMF, as corrinoid reactivation is integral to the function of methyltransferase systems. However, homologs to *mecG* and *mecH* are present elsewhere in the strain DCMF genome (Ga0180325_115323 and Ga0180325_114747, respectively) and were expressed in the proteome. Although they were not significantly more abundant in DCM-amended cells, their expression was above average (i.e., the LFQ Z-score was >0; Supplementary Table S4).

**Figure 4:**
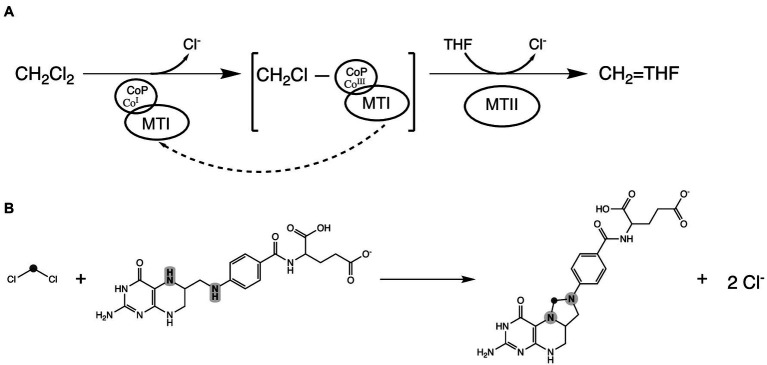
Proposed DCM dechlorination schema in strain DCMF. **(A)** Reduced Co(I) corrinoid protein (CoP), acting in concert with a methyltransferase I protein (MTI), cleaves the first chlorine from DCM, producing a transient CH2Cl-CoP intermediate. Tetrahydrofolate (THF), bound to a methyltransferase II protein (MTII), then attacks the remaining carbon-chlorine bond, ultimately producing 5,10-methylene-tetrahydrofolate. The Co (III) in the corrinoid protein is reduced back to Co (I) by putative reductive activators of corrinoid-dependent enzymes (RACE) proteins, although this may not be required every reaction cycle. **(B)** The overall reaction showing DCM and THF producing 5,10-methylene-tetrahydrofolate. The carbon in DCM is highlighted in dark gray; the N5 and N10 nitrogen heteroatoms in tetrahydrofolate are highlighted in light gray.

Based on the functional annotation of the highly abundant proteins in the DCM-associated gene cluster, we propose a putative mechanism for DCM dechlorination in strain DCMF. Reduced Co(I) in the CoP (Ga0180325_111236, MecB), acting in concert with the MTI protein (Ga0180325_111233, MecE), cleaves one chlorine *via* nucleophilic attack, echoing the role of corrinoid cofactors in reductive dehalogenases (Holliger et al., 1999) and producing a CH_2_Cl-CoP intermediate (Figure 4A). Nitrogen heteroatoms (N5 and N10) in THF, bound by an MTII protein (Ga0180325_111232, MecF), can then attack the transient CH_2_Cl-CoP intermediate in a concerted fashion, eliminating the remaining chloride and cleaving the CoP to form 5,10-methylene-THF (Figure 4). This echoes the model recently proposed by Murdoch et al. (2022). Both schemas suggest an unusual departure from the typical biochemistry of methyltransferase reactions, which do not include chlorinated substituents (Kremp and Müller, 2021).

Proteomic experiments with ‘*Ca.* Dichloromethanomonas elyunquensis’ and *D. formicoaceticum* grown on DCM showed the presence of all genes in the cluster, with the methyltransferases among the most abundant (Kleindienst et al., 2019; Murdoch et al., 2022). However, it is notable that ‘*Ca.* Dichloromethanomonas elyunquensis’ also expressed reductive dehalogenases, which are expected to play a role in DCM dechlorination (Kleindienst et al., 2019). Although putative roles were initially discussed for the reductive dehalogenases (Kleindienst et al., 2019), it is not yet clear how they might act in concordance with the *mec* cassette proteins (Murdoch et al., 2022). If the *mec* cassette alone is responsible for DCM dechlorination, then it is unclear what role the reductive dehalogenases have in ‘*Ca.* Dichloromethanomonas elyunquensis’. Conversely, if reductive dehalogenases are required for DCM dechlorination in ‘*Ca.* Dichloromethanomonas elyunquensis’, then it is not clear what alternative enzymes or pathways for DCM dechlorination exist in strain DCMF and *D. formicoaceticum*, that might act in concert with the *mec* cassette. Previous dual C—Cl isotope analysis implied that dechlorination in *D. formicoaceticum* proceeds *via* a bimolecular nucleophilic substitution pathway (S_N_2), while ‘*Ca.* Dichloromethanomonas elyunquensis’ utilizes a unimolecular (S_N_1) pathway (Chen et al., 2018). Here, the mechanism proposed above for strain DCMF utilizes a S_N_1 reaction, as the nitrogen in folic acid cofactors is a weak nucleophile. However, dual C—Cl isotope analysis and further biochemical characterization of the proteins in the strain DCMF *mec* cassette are required to elucidate the exact dechlorination mechanism.

While data here supports the role of the *mec* cassette in DCM dechlorination, the role of other significantly differentially abundant methyltransferases is less clear. For example, numerous methyltransferase components (20 MTI, 28 MTII and 24 CoP) from strain DCMF were identified in the metaproteome (Supplementary Table S4, “Methyltransferase”). This included 26 of the 82 MttB superfamily methyltransferases (i.e., MTIIs) encoded in the genome (Holland et al., 2019). Seven of these MttB superfamily proteins contain the noncanonical amino acid pyrrolysine (Ga0180325_111271p72, Ga0180325_111278p79, Ga0180325_111485p86, Ga0180325_114321p22, Ga0180325_114324p25, Ga0180325_115207p08, Ga0180325_115773p74). Non-pyrrolysine members of the MttB superfamily are widespread in Bacteria and Archaea and have been shown to encode an increasingly diverse substrate range including glycine betaine (Ticak et al., 2014), proline betaine (Picking et al., 2019), carnitine (Kountz et al., 2020), and γ-butyrobetaine (Ellenbogen et al., 2021). However, pyrrolysine-encoding methyltransferases have thus far only been associated with methanogenesis from methylated amines in Archaea (Ferguson and Krzycki, 1997; Burke et al., 1998; Paul et al., 2000). A limited number of other bacterial genera also encode MttB superfamily genes with the pyrrolysine residue, but to our knowledge, proteomic expression has not previously been observed (Ticak et al., 2014). Further experimental work to elucidate the function of these proteins in strain DCMF could provide insight into a potentially novel role for Pyl-MttB proteins, mirroring the expanding role of their non-pyrrolysine counterparts.

In addition to this, the protein with the greatest LFC in DCM-amended cells sits within the monomethylamine methyltransferase MtmB superfamily (Ga0180325_111810, LFC +11.87). However, monomethylamine was not added to DCM-amended microcosms and would only have been present in glycine betaine-amended microcosms as a metabolic end product, as it cannot be used for growth (Holland et al., 2021). Ga0180325_111810 shares ~30% amino acid sequence identity to other proteins in the monomethylamine methyltransferase superfamily (Supplementary Table S8) but is also lacking the characteristic pyrrolysine residue as described in *Methylosarcina barkeri* MS (Hao et al., 2002). There are two homologs to this protein in *D. formicoaceticum*, albeit with low percentage identities (30.32% to WP_089610783.1, 28.26% to WP_089610108.1), and no homologs in ‘*Ca.* Dichloromethanomonas elyunquensis’. This suggests that perhaps this non-Pyl MtmB superfamily protein has a role outside of monomethylamine demethylation, mirroring the recently reported expanded substrate range of non-Pyl MttB superfamily methyltransferases outlined above.

### Proteins involved in glycine betaine metabolism

Non-Pyl-MttB superfamily methyltransferases were implicated in glycine betaine metabolism in strain DCMF. To our knowledge, this is only the second shotgun proteomic study of a glycine betaine-fermenting bacterium (Visser et al., 2016). In glycine betaine grown strain DCMF cells, Ga0180325_114734 – Ga0180325_114742 were among the most abundant proteins (Figures 1, 2; Supplementary Table S4) and included putative glycine betaine methyltransferases (Table 2). This nine-gene cluster included three MTIs (all within the MttB superfamily), a putative MTII, two CoPs, two glycine betaine transporters, and an N-methylhydantoinase A/oxoprolinase/acetone carboxylase (Table 2). These proteins were all significantly more abundant in cells grown with glycine betaine than DCM (FDR 0.01), with an average LFC of -6.38 (Figure 1; Table 2).

A second cluster of methyltransferase genes was identified amongst the most differentially abundant proteins (Ga0180325_111152 – Ga0180325_111158; Supplementary Table S4). This gene cluster included the two proteins with the largest LFC difference in glycine betaine-amended cells: Ga0180325_111154 (LFC -11.66) and Ga0180325_111152 (LFC -11.01; Figure 1; Supplementary Table S5). These two proteins are both in the MttB superfamily, and their closest characterized homologs are in *Desulfitobacterium hafniense* DCB-2 (Supplementary Table S8). However, it remains unclear whether they act in concert with the other components in this gene cluster, or with those in the cluster mentioned above. Not all proteins in this gene cluster were significantly differentially abundant (the putative CoP Ga0180325_111153 was not), nor were all identified in the metaproteome (Ga0180325_111155, a MtaA/CmuA family protein, was absent). Furthermore, this gene cluster contains only putative MTI, MTII and CoP proteins, with no other genes related to glycine betaine metabolism (e.g., transcriptional regulators or transporters) in the genetic vicinity.

A putative sarcosine reductase gene cluster was also identified: Ga0180325_114795–Ga0180325_114803s encodes a thioredoxin reductase (*trxB*), thioredoxin I (*trxA*), glycine/betaine/sarcosine reductase complex selenoprotein A (*grdA*), protein C (*grdCD)*, and a predicted sarcosine-specific protein B (*grdGF*; Figure 2). A hypothetical protein (Ga0180325_114801) in the middle of the cluster was not identified in the proteome. Excluding that, all proteins were significantly more abundant (FDR 0.01) in glycine betaine amended cells, with an average LFC of -7.58 compared to DCM-amended cells (Figure 1; Table 2; Supplementary Table S5).

Data here supports a previous model for quaternary amine metabolism in strain DCMF that was based on genomic and physiological information (Holland et al., 2021). Briefly, glycine betaine is demethylated to dimethylglycine and then sarcosine; the latter is then reductively cleaved to produce monomethylamine and acetate (Holland et al., 2021). It remains unclear whether any one glycine betaine MTI and CoP pair can catalyze methyl transfer from both glycine betaine and dimethylglycine, as was cautiously suggested for *S. ovata* An4 (Visser et al., 2016). It is possible that that the multiple MTI and CoP subunits in the more complete glycine betaine methyltransferase cluster (Ga0180325_114734–Ga0180325_114742) may be specific to the two substrates, while the singular MTII can transfer the methyl group from either of the CoPs to THF. Alternatively, perhaps the less complete methyltransferase cluster discussed above (Ga0180325_111152–Ga0180325_111158) is specific to dimethylglycine, which would explain why it does not contain a glycine betaine transporter gene.

**Figure 5:**
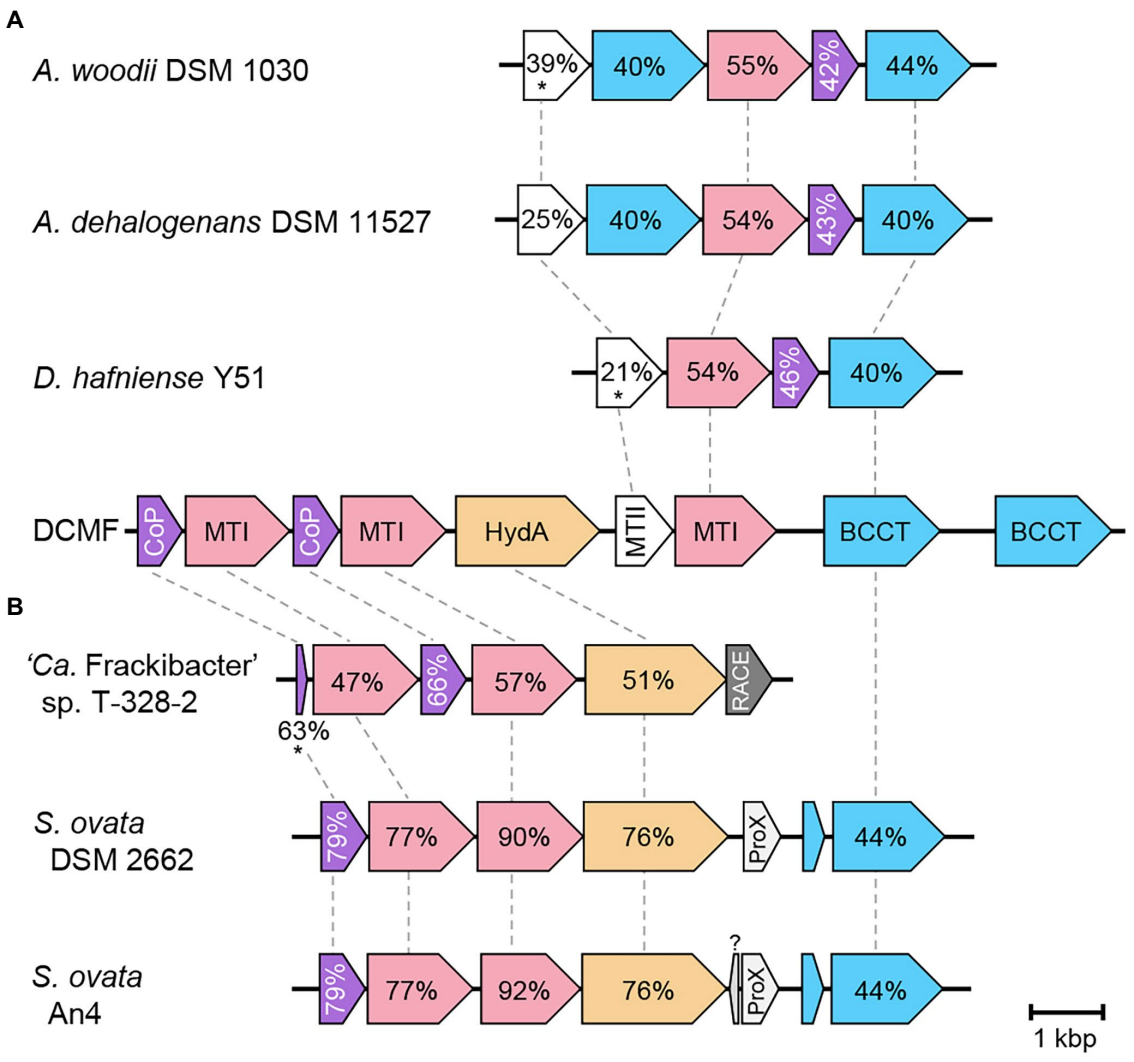
Genetic organization of the glycine betaine methyltransferase gene cluster in strain DCMF and homologs in other glycine betaine-fermenting bacteria. Homologous clusters fell into two distinct groups, with similarity to the second **(A)** or first **(B)** half of the strain DCMF gene cluster. Homologous proteins are linked by dotted lines and percentage amino acid sequence identity to strain DCMF written within. Values in the unlinked BCCT and CoP proteins in Panel A are to the strain DCMF homolog with the highest percentage amino acid sequence identity: Ga0180325_114741 or Ga0180325_114736, respectively, in all cases). Loci shown are: *Acetobacterium woodii* DSM 1030 Awo_c07520 – Awo_07560, *Acetobacterium dehalogenans* DSM 11527 A3KSDRAFT_02713 – A3KSDRAFT_02709, *D. hafniense* Y51 DSY3157 – DSY3154, strain DCMF Ga0180325_114734 – Ga0180325_114742, ‘*Ca.* Frackibacter sp.’ T328-2 AWU54_1980 – AWU54_1975, *Sporomusa ovata* DSM 2662 SOV_3c09370—SOV_3c09310, *S. ovata* An4 SpAn4DRAFT_2140 – SpAn4DRAFT_2133. CoP, corrinoid protein; MTI, methyltransferase I; HydA, hydantoinase; MTII, methyltransferase II; BCCT, betaine/carnitine/choline family transporter; RACE, reductive activator of corrinoid-dependent enzymes; ProX, extracellular glycine betaine ligand binding protein. Asterisks indicate proteins with <30% query coverage of the strain DCMF homolog.

Comparison of the complete glycine betaine methyltransferase gene cluster with homologs from other glycine betaine-fermenting bacteria revealed two broad gene cluster architectures, which has not been previously reported. Experimentally proven glycine betaine methyltransferase operons in *A. woodii* DSM 1030 (Lechtenfeld et al., 2018) and *Desulfitobacterium hafniense* Y51 (Ticak et al., 2014) demonstrate genomic synteny, along with a homologous gene cluster identified in *A. dehalogenans* DSM 11527 (Figure 5A). This region is echoed in the second half of the glycine betaine gene cluster identified in strain DCMF (Figure 5). In contrast, the glycine betaine fermenting bacteria *S. ovata* DSM 2662, *S. ovata* An4, and ‘*Candidatus* Frackibacter sp.’ T328-2 all encode syntenic regions to the first half of the strain DCMF cluster (Figure 5B). For the *S. ovata* strains, these genes differ from those suggested to be responsible for glycine betaine demethylation in previous works, offering new candidates for further study (Visser et al., 2016; Lechtenfeld et al., 2018). These genes suggested here were among the numerous abundant methyltransferases during glycine betaine metabolism by *S. ovata* An4 (Visser et al., 2016), while proteomic studies of *A. woodii* DSM 1030 and *D. hafniense* Y51 have not yet been reported. Strain DCMF similarly expressed a range of methyltransferases with both DCM and glycine betaine-amended cultures, and investigation into the apparent redundancy of these proteins may yield interesting results.

It is worth noting that, while the role of DCM in the broader global carbon budget has only recently been reconsidered with increased significance (Hossaini et al., 2017; Murdoch et al., 2022), the links between glycine betaine and climate-active gasses have been reported for decades. In anoxic subsurface environments, it can be microbially transformed to methylamines (Naumann et al., 1983; King, 1984; Möller et al., 1986; Zindel et al., 1988; Heijthuijsen and Hansen, 1989; Mouné et al., 1999; Daly et al., 2016), which are then metabolized almost exclusively by methanogens, generating the potent greenhouse gas methane (Oremland et al., 1982; King, 1984, 1988; Oren, 1990; Ollivier et al., 1994; Daly et al., 2016). Additionally, some species of *Methanococcoides* can also utilize glycine betaine directly for methanogenesis (Watkins et al., 2014). The environmental relevance of strain DCMF therefore extends beyond DCM-contaminated sites, as the pathways involved in both DCM and glycine betaine degradation affect the flux of climate-active gasses from anoxic, subsurface environments.

### Metaproteogenomic insights into the non-dechlorinating community

None of the cohabitant MAGs contain a complete Wood–Ljungdahl pathway, which is consistent with a previous observation that culture DFE cannot grow autotrophically (Holland et al., 2021). Instead, the MAGs encode genes indicative of oxidative glycolysis for central carbon metabolism. Additionally, the cohabitant MAGs do not encode any corrinoid-dependent methyltransferases or corrinoid proteins, nor homologs to any of the genes in the *mec* cassette. However, DFE-SYN encoded multiple glycine/betaine/sarcosine reductase genes, including one cluster with all components of a reductase complex and thioredoxin (DFE_SYN_02509–DFE_SYN_02516). The reductase complex component B subunits gamma (DFE_SYN_02511) and alpha/beta (DFE_SYN_02514) were both identified in the metaproteome (Supplementary Table S4). While substrate specificity of this reductase could not be predicted based on sequence similarity to known enzymes (Supplementary Figure S3), there are no published reports of any Synergistetes utilizing glycine betaine or sarcosine, and both components were more highly expressed in DCM-amended cultures than those with glycine betaine (Supplementary Table S4). This suggests that they may be specific to glycine, rather than sarcosine or glycine betaine. Overall, metagenomic data supported previous experimental data suggesting that the cohabitant bacterial lineages are not able to consume any of the primary substrates added to culture DFE, but rather feed on expired cellular material, i.e., necromass (Holland et al., 2021).

In addition to this, we hypothesize that at least some of the cohabiting lineages provide a benefit to strain DCMF, given their persistence despite repeated attempts to isolate the bacterium (Holland et al., 2019, 2021). Cohabiting bacteria can provide various benefits to keystone species, including production of amino acids or essential cofactors for which the other is auxotrophic (e.g., Yan et al., 2012; Embree et al., 2015), or removing nitrogen-rich wastes that might otherwise accumulate to toxic levels (e.g., Christie-Oleza et al., 2017). However, in culture DFE, the exact nature of the syntrophic partnership between strain DCMF and the cohabiting bacteria remains unclear.

Nonetheless, lineages identified in culture DFE—*Cupidesulfovibrio*, *Bacteroidales*, *Spirochaetes/Treponematales*, *Synergistetes*—have previously been associated with hydrocarbon and organohalide-degrading cultures (Duhamel and Edwards, 2006; Kleinsteuber et al., 2008; Strąpoć et al., 2011; Taubert et al., 2012; Dong et al., 2018), where some reports have suggested that they persist *via* necromass recycling (Kleinsteuber et al., 2012; Lee et al., 2012; Taubert et al., 2012; Dong et al., 2018). Microbial necromass utilization is increasingly being considered as an important contributor to subsurface nutrient cycling (Simpson et al., 2007; Liang et al., 2011). At contaminated sites in particular, it may enhance bioremediation, as the production of hydrogen, acetate, and ethanol can both stimulate microbial blooms or serve as secondary substrates for co-metabolism (Horvath, 1972; Wrighton et al., 2014). Outside of necromass studies, similarly “self-feeding” mixed cultures have been described for dechlorination of trichloromethane and DCM (Wang et al., 2022), chlorobenzene (Liang et al., 2013), and 3-chlorobenzoate (Dolfing, 1986; Dolfing and Tiedje, 1987). Consumption of dead biomass can also remineralize or liberate important nutrients including nitrogen, phosphorous, trace elements, and cobalamins (Head et al., 2006; Christie-Oleza et al., 2017). Following in this theme are calls from other authors for the use of mixed consortia, rather than monocultures for biotechnological processes (Giri et al., 2020; Borchert et al., 2021). Diverse communities are more robust against environmental and ecological disturbances and have increased functionality due to synergistic interspecies interactions (Chiu et al., 2014; Pande and Kost, 2017; Giri et al., 2020; Pascual-García et al., 2020). Understanding microbial interactions in culture DFE, in which a chlorinated one-carbon compound has sustained a stable community for 10 years, can reveal new insights into syntrophic community dynamics and could aid the development of more robust mixed cultures for *in situ* bioremediation applications.

## Conclusion

Proteomic study of the DCM-fermenting bacterium in culture DFE, ‘*Ca.* Formimonas warabiya’ strain DCMF, supports the role of a methyltransferase system encoded by the *mec* cassette in DCM dechlorination. The *mec* cassette encodes candidate genes for tracking anaerobic DCM metabolism *in situ* at contaminated sites, and this work lays the foundation for biochemical/structural characterization of DCM dechlorinating enzymes. Proteogenomic evidence for a putative glycine betaine methyltransferase in strain DCMF also added to our limited knowledge regarding the fate of this environmentally important compound in anoxic subsurface environments. Furthermore, analysis of metaproteogenomic data from the cohabiting linegaes in culture DFE supported the previous hypothesis that they are not involved in primary substrate metabolism, but rather persist *via* metabolism of necromass from spent cells.

## Data availability statement

The datasets presented in this study can be found in online repositories. The names of the repository/repositories and accession number(s) can be found in the article/Supplementary material.

## Author contributions

SH, MM, and ML conceived the study. SH performed the experiments, wrote the manuscript, and produced the figures. SH and HE analyzed the data. XV-C and RE advised and assisted with metagenomic assembly and analysis. RE and MM contributed the resources. All authors contributed to the article and approved the submitted version.

## Funding

SH was supported by an Australian Government Research Training Program Scholarship. XV-C acknowledges support from the New South Wales State Government RAAP scheme and the National Collaborative Research Infrastructure Strategy. RE was funded by the Australian Research Council (ARC LP160100610).

## Conflict of interest

The authors declare that the research was conducted in the absence of any commercial or financial relationships that could be construed as a potential conflict of interest.

## Publisher’s note

All claims expressed in this article are solely those of the authors and do not necessarily represent those of their affiliated organizations, or those of the publisher, the editors and the reviewers. Any product that may be evaluated in this article, or claim that may be made by its manufacturer, is not guaranteed or endorsed by the publisher.
