# Supplementary material for: Metaproteomics reveals methyltransferases implicated in dichloromethane and glycine betaine fermentation by ‘Candidatus Formimonas warabiya’ strain DCMF
Source: Front Microbiol. 2022 Dec 7;13:1035247. doi: 10.3389/fmicb.2022.1035247 (PMC9768040; doi:10.3389/fmicb.2022.1035247)
Supplement: Supplementary file 1 [file Data_Sheet_1.PDF]

Supplementary Material

1 Supplementary Discussion

1.1 Survival of non-DCM fermenting bacteria in culture DFE

The presence of numerous electron transport chain genes in the DFE-LEN, DFE-NIT and DFE-BAC MAGs suggested that they are capable of anaerobic respiration. These include various genes for complex I, a succinate dehydrogenase/fumarate reductase, and an F-type ATP synthase (Supplementary Table 3). Of these, only two units of the DFE-NIT ATP synthase were identified in the proteome (DFE_NIT_02269 and DFE_NIT_02271; Supplementary Table 4). It appears more likely that these taxa utilise fermentative metabolisms within culture DFE, as discussed below. Conversely, DFE-SPI, DFE-TRE1, and DFE-TRE2 appeared to only be capable of fermentative metabolisms. All three MAGs encoded V-type ATP synthases but no other electron transport chain machinery, and the latter two encoded a wider variety of ABC transporters (84 unique genes in DFE-TRE1 and 41 in DFE-TRE2; Supplementary Table 3).

The vast majority of cohabitant bacterial proteins identified in the metaproteome belonged to DFE-SYN, one of the most complete MAGs (94.45%) and abundant cohabitants, according to previous community analysis of culture DFE (Holland et al., 2021). This lineage is expected to persist via amino acid fermentation in culture DFE (Supplementary Figure 3). *Synergistales* are widely distributed throughout diverse environments in nature (Hugenholtz et al., 1998; Vartoukian et al., 2007), with members of the phylum *Synergistetes* found in 90% of anaerobic environments surveyed by Godon *et al*, although typically only a minor constituent of the community (Godon et al., 2005). *Synergistales* are well-known amino acid fermenters (Jumas-Bilak et al., 2007; Pitluck et al., 2010), and some species are also saccharolytic (Vartoukian et al., 2007), meaning they likely capitalize on the amino acids and dicarboxylates present in culture DFE (Supplementary Figure 3). Indeed, ABC transporters for branched chain amino acids and C4-dicarboxylates were among the most highly abundant proteins expressed by DFE-SYN (e.g. DFE_SYN_02394, DFE_SYN_02559, DFE_SYN_02455, DFE_SYN_02307). The MAG also encoded 19 genes for the citrate cycle and a glutamate dehydrogenase – important for linking amino acids with the tricarboxylic acid cycle – was highly abundant in the metaproteome (DFE_SYN_01178).

Similar, DFE-TRE1 and DFE-TRE2 are also predicted to persist in culture DFE by utilising amino acids (Supplementary Figure 3). The GTDB-named family *Treponematales* are a subset of the *Spirochaetales* in NCBI taxonomy. Spirochaetes are commonly detected in anoxic environments contaminated with hydrocarbons and organohalides (Einsiedl et al., 2015; Tan et al., 2015) and a recent report demonstrated their role as necromass recyclers in such ecosystems (Dong et al., 2018). Within the DFE community, both DFE-TRE1 and DFE-TRE2 appear to be more versatile scavengers than DFE-SYN, as they encode transport proteins for a wide range of substrates, including monosaccharides, oligosaccharides, amino acids, and peptides (Supplementary Table 3). Several of these were expressed in the metaproteome (Supplementary Table 4). For DFE-TRE1, assigned to the genus *Rectinema*, this is congruent with the sole other cultured member of the genus, *R. subterraneum*, which was shown to grow with mono-, di- and polysaccharides as electron donors, as well as hydrogen (Momper et al., 2020). DFE-TRE2 also encoded an NADP-dependent [FeFe] Group A1 hydrogenase (DFE_TRE2_01688, DFE_TRE2_01689, DFE-TRE2_01690; the latter two

components were both expressed in the metaproteome), suggesting that it may produce hydrogen from fermentation of amino acids and other necromass constituents (Supplementary Figure 3). Fermentation and hydrolysis of these compounds may also produce alcohols and short-chain fatty acids (Dong et al., 2018), which could in turn be utilized by other cohabitant bacteria.

The DFE-LEN MAG was near complete (95.43%) and was second-most abundant of the cohabitants in the metaproteome (60 proteins). An earlier release of the GTDB (r95) categorised this MAG (now an unclassified *Bacteriodales*) in the family *Lentimicrobiaceae,* a taxon within the phylum Bacteroidetes in which the sole cultured representative, *Lentimicrobium saccharophilum*, was shown to ferment a range of carbohydrates (Sun et al., 2016). While the DFE-LEN did encode a near-complete electron transport chain, none of these genes were expressed in the metaproteome, indicating that it may indeed defer to fermentation for survival within culture DFE. Distinguishing DFE-LEN from the amino acid fermenting lineages discussed above, the bacterium may utilise carbohydrates within culture DFE (Supplementary Figure 3), as it encoded the highest number of CAZymes and predicted extracellular proteins, which is important for degrading large biopolymers inherent in microbial biomass (Christie-Oleza et al., 2015). This may assist other organisms in the DFE culture by making larger molecules more metabolically available. The expression of multiple proteins that are homologous to Rag/Sus nutrient uptake outer membrane proteins (e.g. DFE_LEN_00454, DFE_LEN_00595, DFE_LEN_02136, DFE_LEN_02137) indicates that DFE-LEN is also likely able to import larger molecules resulting from the extracellular degradation of proteins (Hall et al., 2005) and carbohydrates (Cho and Salyers, 2001). The presence of an NADP-reducing FeFe Group A1 hydrogenase in the MAG (*hndANCD*), one subunit of which was found in the metaproteome (DFE_LEN_01392), indicates that it may produce hydrogen during fermentation (Supplementary Figure 3).

DFE-NIT was classified as *Cupidesulfovibrio*, a recently proposed novel genus derived from *Desulfovibrio vulgaris*, and *Desulfovibrio oxamicus* (Wan et al., 2021). Species in the sulfate-respiring genera *Desulfovibrio* and *Cupidesulfovibrio* have a broad substrate range and have been associated with contaminated environments both as a primary degrader (Löffler et al., 2005; Kleinsteuber et al., 2008) and a synergistic cohabitant consuming fermentation products of other organisms (Müller et al., 2009; Taubert et al., 2012; Tan et al., 2015). Previous work demonstrated the enrichment of a *Desulfovibrio* sp. to apparent purity in peptone-amended sub-cultivations of DFE (Holland et al., 2021), showing that the lineage within culture DFE is capable of a fermentative, proteolytic metabolism (Supplementary Figure 3). However, the DFE-NIT MAG also encoded a near complete electron transport chain, as well as genes for nitrogen fixation and dissimilatory sulfate reduction (Supplementary Table 3). Proteins for sulfate reduction (AprA, DFE_NIT_00092; AprB, DFE_NIT_00091; DsvA, DFE_NIT_01880; DsvB, DFE_NIT_01881) and subunits for a periplasmic [NiFe] Group 1a and 1b hydrogenase (both groups encode unidirectional hydrogen-uptake hydrogenases for respiration; DFE_NIT_02699, DFE_NIT_02700, DFE_NIT_02702, DFE_NIT_02703) were also identified in the metaproteome. While this suggests that DFE-NIT could be utilising hydrogen produced by other fermenters in the culture as an electron donor for sulfate reduction, there is no obvious source of sulfate in the medium, except perhaps from sulfur-containing amino acid degradation. Overall, however, it seems more likely that DFE-NIT persists via the demonstrated proteolytic metabolism in the DFE community. It is possible that the proteins for sulfate reduction are constitutively expressed, even in the absence of sulfate, as was shown for *Desulfovibrio desulfuricans* strain 27774 (Marietou et al., 2009).

2 Supplementary Figures

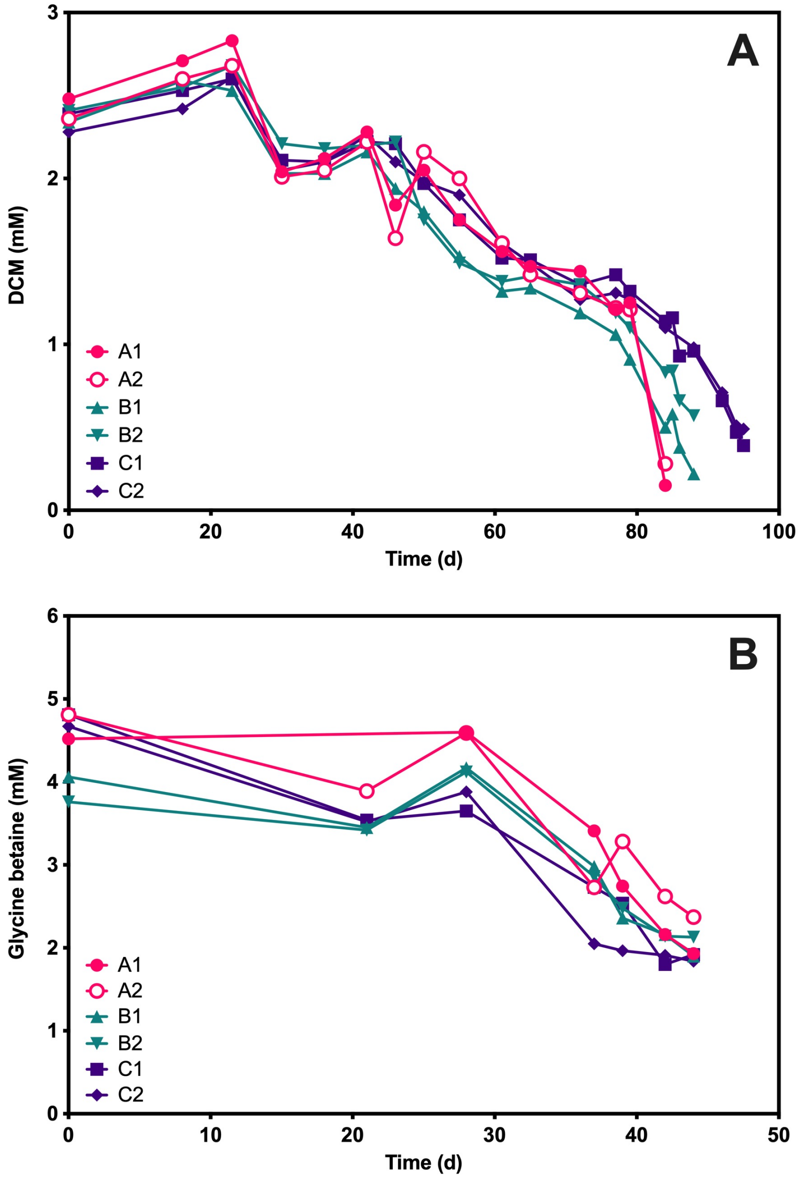

Supplementary Figure 1. DCM (A) and glycine betaine (B) degradation in cultures used for metaproteomic analysis. Replicates (200 ml) were combined in duplicate to generate triplicate samples for analysis (A1+A2, B1+B2, C1+C2).

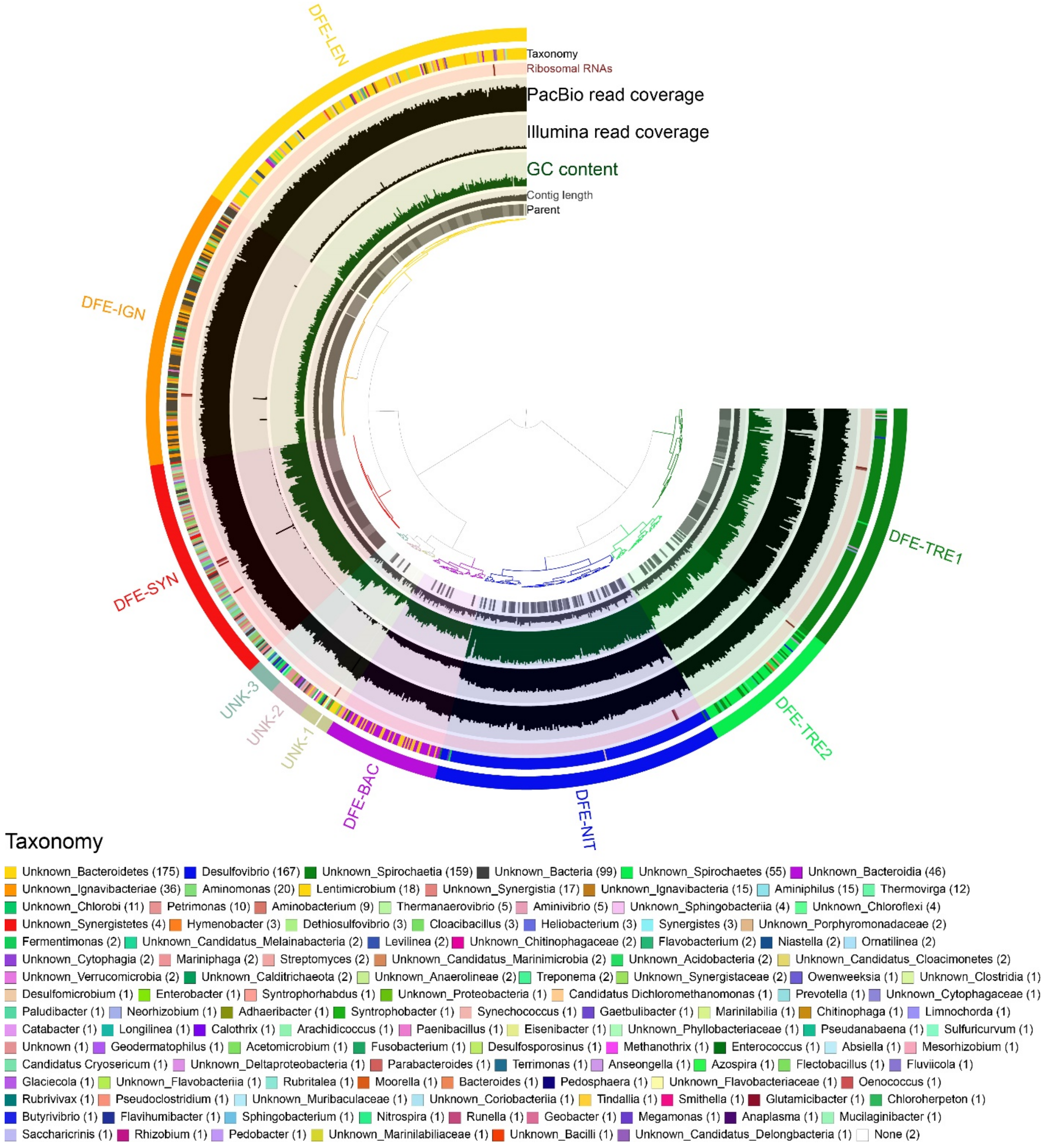

Supplementary Figure 2. Genome-centric metagenomic analysis of culture DFE. The assembled and frameshift-corrected DFE metagenome was binned manually in Anvi'o, informed by the PacBio long read sequencing coverage, Illumina short read sequencing coverage, and taxonomic assignment of individual contigs. Rings have the following scales: Contig length 0 – 43,100 bp; GC content 30 – 75%; Illumina read coverage 0 – 1.68 on a log_{10} scale (equivalent to 0 – 46.54); PacBio read coverage 0 – 1.88 on a log_{10} scale (equivalent to 0 – 74.38); Ribosomal RNAs are present in contigs marked by dark red lines. Taxonomy for each contig was determined in Kaiju v1.7.2 and is marked as indicated in the legend above.

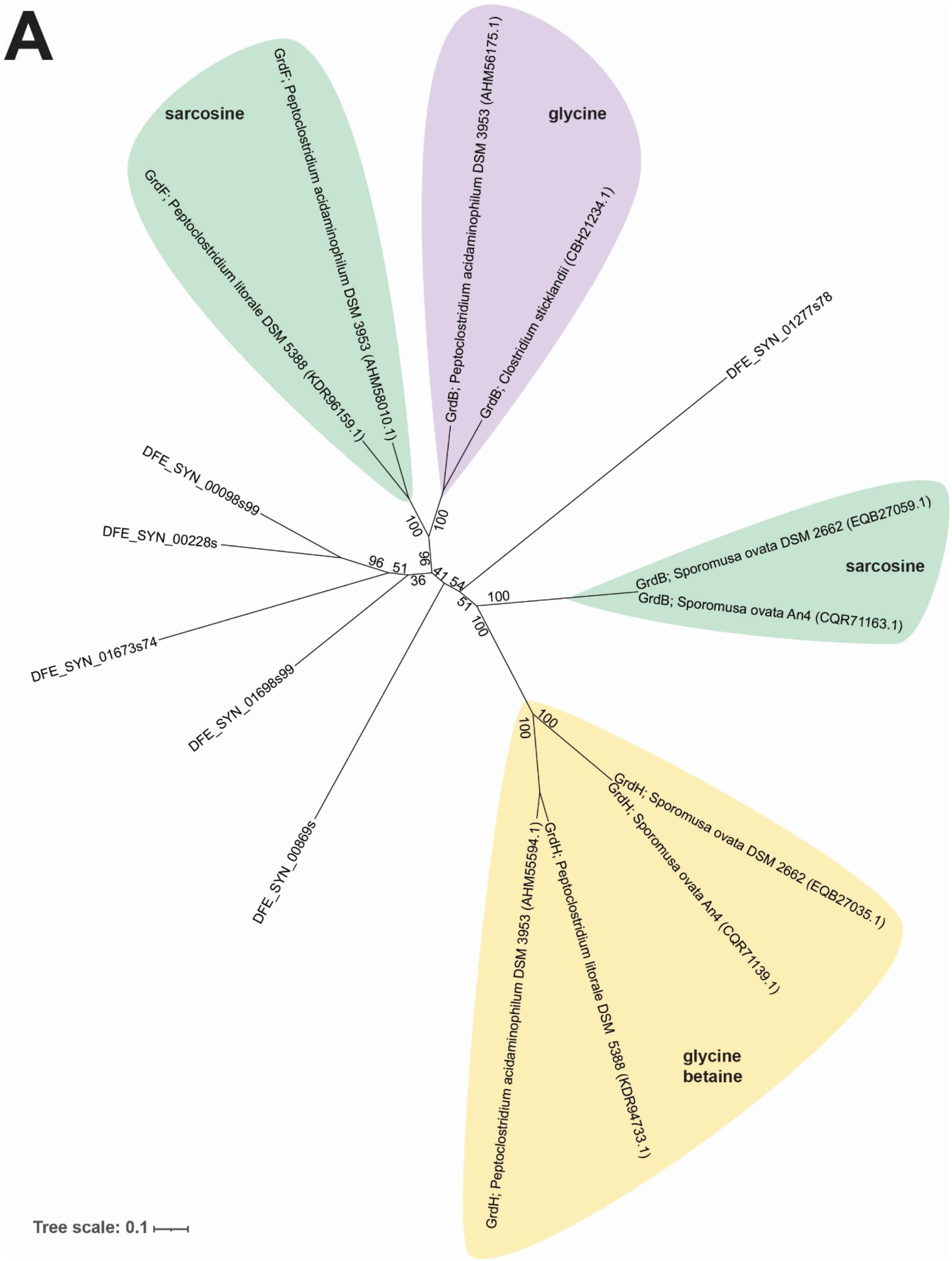
A
sarcosine
GrdF; Peptoclostridium acidaminophilum DSM 3953 (AHM58010.1)
GrdF; Peptoclostridium litorale DSM 5388 (KDR96159.1)
glycine
GrdB; Peptoclostridium acidaminophilum DSM 3953 (AHM56175.1)
GrdB; Clostridium sticklandii (CBH21234.1)
DFE_SYN_01277s78
DFE_SYN_00098s99
DFE_SYN_00228s
DFE_SYN_01673s74
DFE_SYN_01698s99
DFE_SYN_00869s
sarcosine
GrdB; Sporomusa ovata DSM 2662 (EQB27059.1)
GrdB; Sporomusa ovata An4 (CQR71163.1)
glycine betaine
GrdH; Sporomusa ovata DSM 2662 (EQB27035.1)
GrdH; Sporomusa ovata An4 (CQR71139.1)
GrdH; Peptoclostridium acidaminophilum DSM 3953 (AHM55594.1)
GrdH; Peptoclostridium litorale DSM 5388 (KDR94733.1)
100
100
96
51
96
41
54
36
100
51
100
100
100
Tree scale: 0.1

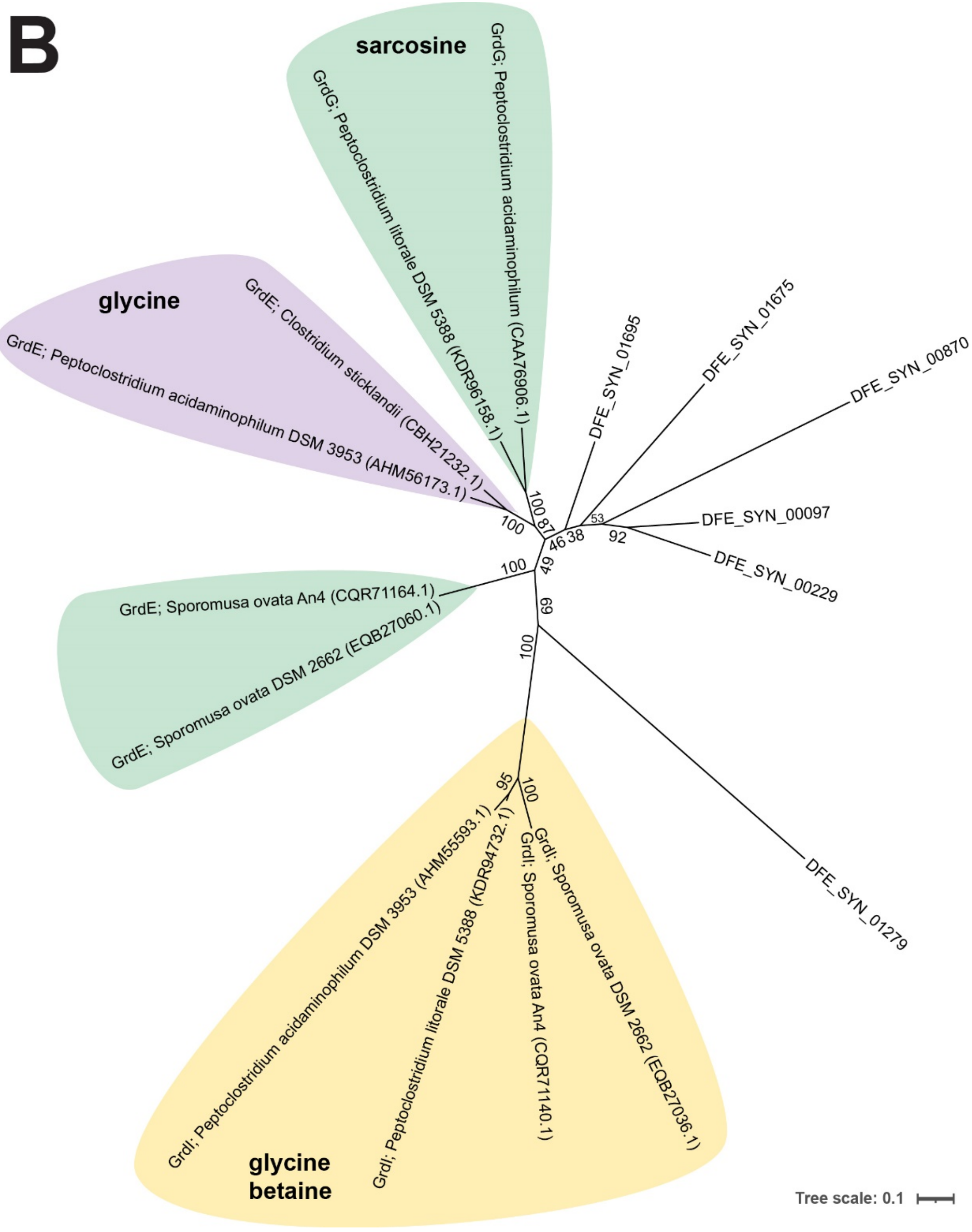

Supplementary Figure 3. Unrooted Maximum Likelihood trees of predicted glycine/betaine/sarcosine reductase complex B proteins from DFE-SYN with those of known function from other bacteria. An attempt was made to predict the substrate specificity of **A.** predicted GrdBFH (selenocysteine-containing) and **B.** GrdEGI proteins from DFE-SYN. Full length amino acid sequences from DFE-SYN were aligned with those of known substrate specificity with MUSCLE in UGENE v1.32 (Okonechnikov et al., 2012). An unrooted Maximum Likelihood tree (1000 bootstraps) was inferred by IQ-Tree v1.6.1 using Model Finder (Nguyen et al., 2015; Kalyaanamoorthy et al., 2017) and visualized in iTOL v6.6 (Letunic and Bork, 2021).

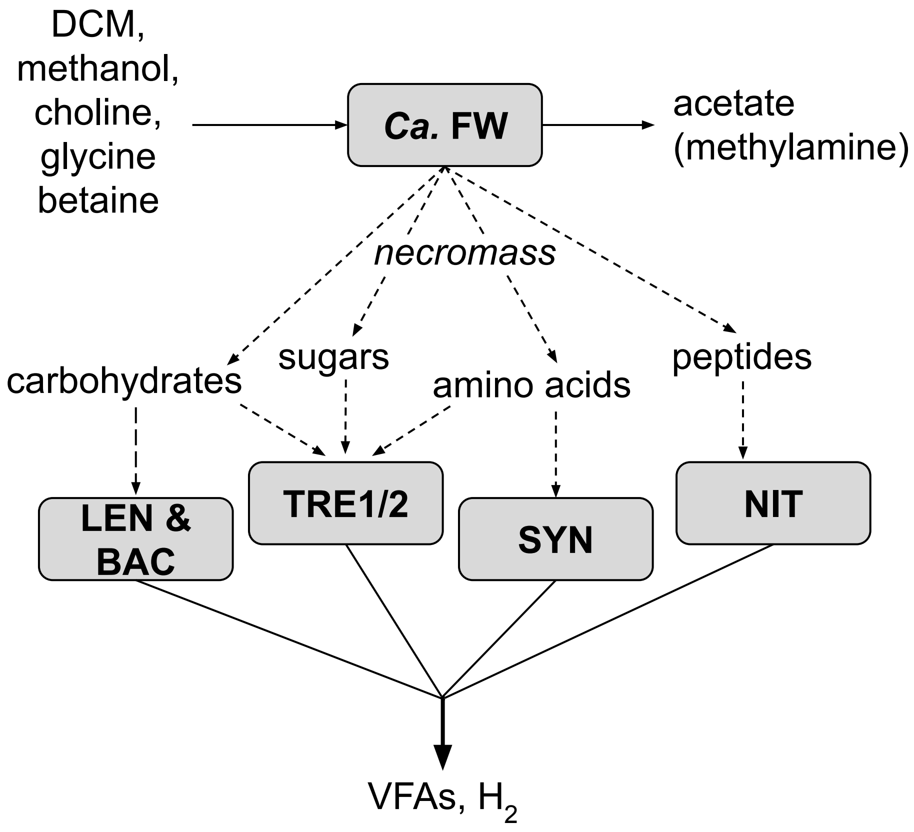

Supplementary Figure 4. Putative community interactions in culture DFE. Cohabiting lineages are proposed to persist in the culture via necromass utilisation, i.e., consumption of carbohydrates, sugars, amino acids, and peptides resulting from expired DCMF cells. VFAs, volatile fatty acids.

3 Supplementary References

Cho, K. H., and Salyers, A. A. (2001). Biochemical analysis of interactions between outer membrane proteins that contribute to starch utilization by *Bacteroides thetaiotaomicron*. *J Bacteriol* 183, 7224–7230. doi: 10.1128/JB.183.24.7224-7230.2001.

Christie-Oleza, J. A., Scanlan, D. J., and Armengaud, J. (2015). "You produce while I clean up", a strategy revealed by exoproteomics during *Synechococcus-Roseobacter* interactions. *Proteomics* 15, 3454–3462. doi: 10.1002/pmic.201400562.

Dong, X., Greening, C., Brüls, T., Conrad, R., Guo, K., Blaskowski, S., et al. (2018). Fermentative Spirochaetes mediate necromass recycling in anoxic hydrocarbon-contaminated habitats. *ISME J* 12, 2039–2050. doi: 10.1038/s41396-018-0148-3.

Einsiedl, F., Pilloni, G., Ruth-Anneser, B., Lueders, T., and Griebler, C. (2015). Spatial distributions of sulphur species and sulphate-reducing bacteria provide insights into sulphur redox cycling and biodegradation hot-spots in a hydrocarbon-contaminated aquifer. *Geochim Cosmochim Acta* 156, 207–221. doi: 10.1016/j.gca.2015.01.014.

Godon, J. J., Morinière, J., Moletta, M., Gaillac, M., Bru, V., and Delgènes, J. P. (2005). Rarity associated with specific ecological niches in the bacterial world: The "Synergistes" example. *Environ Microbiol* 7, 213–224. doi: 10.1111/j.1462-2920.2004.00693.x.

Hall, L. M. C., Fawell, S. C., Shi, X., Faray-Kele, M. C., Aduse-Opoku, J., Whiley, R. A., et al. (2005). Sequence diversity and antigenic variation at the rag locus of *Porphyromonas gingivalis*. *Infect Immun* 73, 4253–4262. doi: 10.1128/IAI.73.7.4253-4262.2005.

Holland, S. I., Ertan, H., Montgomery, K., Manefield, M. J., and Lee, M. (2021). Novel dichloromethane-fermenting bacteria in the *Peptococcaceae* family. *ISME J* 15, 1709–1721. doi: 10.1038/s41396-020-00881-y.

Hugenholtz, P., Goebel, B. M., and Pace, N. R. (1998). Erratum: Impact of culture-independent studies on the emerging phylogenetic view of bacterial diversity (Journal of Bacteriology (1998) 180:18 (4765-4774)). *J Bacteriol* 180, 6793.

Jumas-Bilak, E., Carlier, J. P., Jean-Pierre, H., Citron, D., Bernard, K., Damay, A., et al. (2007). *Jonquetella anthropi* gen. nov., sp. nov., the first member of the candidate phylum "*Synergistetes*" isolated from man. *Int J Syst Evol Microbiol* 57, 2743–2748. doi: 10.1099/ijs.0.65213-0.

Kalyaanamoorthy, S., Minh, B. Q., Wong, T. K. F., von Haeseler, A., and Jermiin, L. S. (2017). ModelFinder: fast model selection for accurate phylogenetic estimates. *Nat Methods* 14, 587–589. doi: 10.1038/nmeth.4285.

Kleinsteuber, S., Schleinitz, K. M., Breitfeld, J., Harms, H., Richnow, H. H., and Vogt, C. (2008). Molecular characterization of bacterial communities mineralizing benzene under sulfate-reducing conditions. *FEMS Microbiol Ecol* 66, 143–157. doi: 10.1111/j.1574-6941.2008.00536.x.

Letunic, I., and Bork, P. (2021). Interactive tree of life (iTOL) v5: An online tool for phylogenetic tree display and annotation. *Nucleic Acids Res* 49, W293–W296. doi: 10.1093/nar/gkab301.

Löffler, F. E., Sanford, R. a, and Ritalahti, K. M. (2005). Enrichment, cultivation, and detection of reductively dechlorinating bacteria. *Methods Enzymol* 397, 77–111. doi: 10.1016/S0076-6879(05)97005-5.

Marietou, A., Griffiths, L., and Cole, J. (2009). Preferential reduction of the thermodynamically less favorable electron acceptor, sulfate, by a nitrate-reducing strain of the sulfate-reducing bacterium *Desulfovibrio desulfuricans* 27774. *J Bacteriol* 191, 882–889. doi: 10.1128/JB.01171-08.

Momper, L., Semler, A., Lu, G. S., Miyazaki, M., Imachi, H., and Amend, J. P. (2020). *Rectinema subterraneum* sp. nov, a chemotrophic spirochaete isolated from the deep terrestrial subsurface. *Int J Syst Evol Microbiol* 70, 4739–4747. doi: 10.1099/ijsem.0.004339.

Müller, S., Vogt, C., Laube, M., Harms, H., and Kleinsteuber, S. (2009). Community dynamics within a bacterial consortium during growth on toluene under sulfate-reducing conditions. *FEMS Microbiol Ecol* 70, 586–596. doi: 10.1111/j.1574-6941.2009.00768.x.

Nguyen, L. T., Schmidt, H. A., von Haeseler, A., and Minh, B. Q. (2015). IQ-TREE: A fast and effective stochastic algorithm for estimating maximum-likelihood phylogenies. *Mol Biol Evol* 32, 268–274. doi: 10.1093/molbev/msu300.

Okonechnikov, K., Golosova, O., Fursov, M., and the UGENE team (2012). Unipro UGENE: A unified bioinformatics toolkit. *Bioinformatics* 28, 1166–1167. doi: 10.1093/bioinformatics/bts091.

Pitluck, S., Yasawong, M., Held, B., Lapidus, A., Nolan, M., Copeland, A., et al. (2010). Non-contiguous finished genome sequence of *Aminomonas paucivorans* type strain (GLU-3 T). *Stand Genomic Sci* 3, 285–293. doi: 10.4056/sigs.1253298.

Sun, L., Toyonaga, M., Ohashi, A., Tourlousse, D. M., Matsuura, N., Meng, X. Y., et al. (2016). *Lentimicrobium saccharophilum* gen. nov., sp. nov., a strictly anaerobic bacterium representing a new family in the phylum *Bacteroidetes*, and proposal of *Lentimicrobiaceae* fam. nov. *Int J Syst Evol Microbiol* 66, 2635–2642. doi: 10.1099/ijsem.0.001103.

Tan, B., Jane Fowler, S., Laban, N. A., Dong, X., Sensen, C. W., Foght, J., et al. (2015). Comparative analysis of metagenomes from three methanogenic hydrocarbon-degrading enrichment cultures with 41 environmental samples. *ISME J* 9, 2028–2045. doi: 10.1038/ismej.2015.22.

Taubert, M., Vogt, C., Wubet, T., Kleinsteuber, S., Tarkka, M. T., Harms, H., et al. (2012). Protein-SIP enables time-resolved analysis of the carbon flux in a sulfate-reducing, benzene-degrading microbial consortium. *ISME J* 6, 2291–2301. doi: 10.1038/ismej.2012.68.

Vartoukian, S. R., Palmer, R. M., and Wade, W. G. (2007). The division "*Synergistes*." *Anaerobe* 13, 99–106. doi: 10.1016/j.anaerobe.2007.05.004.

Wan, Y. Y., Luo, N., Liu, X.-L., Lai, Q.-L., and Goodfellow, M. (2021). *Cupidesulfovibrio liaohensis* gen. nov., sp. nov., a novel sulphate-reducing bacterium isolated from an oil reservoir and reclassification of *Desulfovibrio oxamicus* and *Desulfovibrio termitidis* as *Cupidesulfovibrio oxamicus* comb. *Int J Syst Evol Microbiol* 71, 1–9. doi: 10.1099/ijsem.0.004618.
